# Supplementary material for: Virological non-suppression among adult males attending HIV care services in the fishing communities in Bulisa district, Uganda
Source: PLoS One. 2023 Oct 19;18(10):e0293057. doi: 10.1371/journal.pone.0293057 (PMC10586650; doi:10.1371/journal.pone.0293057)
Supplement: S1 File — (PDF) [file pone.0293057.s001.pdf]

AGREEMENT
between
Mr. Ignatius Senteza, *Gilead Masters* Research Fund Scholarship Grantee
and Infectious Diseases Institute

THIS AGREEMENT is made between Mr. Ignatius Senteza (hereinafter 'Masters Student') and the Infectious Diseases Institute (hereinafter 'IDI'), a Ugandan company registered as a non-governmental organization, having its principal place of business at College of Health Sciences of Makerere University, Mulago Hospital Complex, P.O. Box 22418, Kampala, Uganda.

WHEREAS the IDI operates a Research, HIV/AIDS Prevention Care and Treatment clinic, laboratory, and training center with the mission: to strengthen health systems in Africa with a strong emphasis on infectious diseases through research and capacity development

and

WHEREAS the Student has been selected as recipient of the *Gilead Masters* Studentship (hereinafter 'Studentship') on the basis of:

- Enrollment in the Makerere University Master's Degree Program
- Outstanding progress in the Master's program
- Demonstrated interest in research
- Letters of recommendation
- A written statement of interest in "Improving Care in Infectious Diseases".
- A personal interview with the selection committee.

IT IS THEREFORE AGREED THAT:

1. Aims of the *Gilead Masters* Studentship Grant

The *Gilead Masters Research Fund* Studentship Grant seeks to support and mentor outstanding postgraduate students in the College of Health Sciences to become excellent clinicians and researchers; with the intention of inclining them towards the pursuit of an academic career in management and prevention of Infectious Diseases.

The Studentship shall be in the form of financial and supervisory support to the Masters student. The support is cognizant of the existing Masters Semester curriculum and shall seek to augment it.

It is expected that, after completion of the Studentship, the Masters Student will have made significant progress towards, or successfully completed, the requirements for the Makerere University Master's Program including the research dissertation.

Student Expectations:

1. Submit quarterly reports to IDI Capacity Building Unit.

2. Complete Good Clinical Practice (GCP) and Human Research Subjects (HRS) courses and submit valid certificates for these.
3. Complete the Individual Development Plan as a requirement for all sponsored scholars at **http://myidp.sciencecareers.org/** and submit an IDP summary.
4. Submit an abstract to an international meeting
5. Before submitting the study proposal to the School of Public Health, the student must submit the proposal to the IDI Scientific Review Committee and follow others SRC requirements as stated in the "IDI scholars SOPs for study proposal submission" document.
6. Present the research project to the IDI research forum
7. Write thesis
8. Submit a manuscript to a peer-reviewed journal.
9. The scholar must acknowledge Support for research was provided by "Gilead foundation" through the Gilead Infectious Diseases scholarship program of the Infectious Diseases Institute Limited (IDI) of Makerere University as the funding body in all research publications, abstracts and presentations that are made while s/he receives support from Gilead.as the funding body in all research publications, abstracts and presentations that are made while s/he receives support from Gilead.
10. Mentor written appraisals 2 times/year in the last year
11. Submit report at the end of studentship report

The student is also expected to attend IDI research forums whenever it is possible, and to interact with the other IDI and Academy scholars

2. Studentship Structure

As noted above the student shall be enrolled in a structured semester system. The Student is expected to maintain an exemplary standard of performance in the Master's program, with a minimum composite Grade Point Average (GPA) of **4.0** at the end of each semester.

3. Assessment and Examination

The student will be formally assessed by the School of Public Health and the IDI Head of Research. Assessments shall coincide with the end of each semester in order to include the formal student assessments carried out by the Makerere University College of Health Sciences.

- The Student will be assigned an IDI supervisor.
- The student has to submit progress quarterly reports to the IDI Capacity Building Unit.
- In the course of the year the student shall meet with the IDI supervisors on at least 3 occasions to receive feedback and mentorship as needed.

4. Financial Support

In consideration of the student's enrollment in a full time program of study the IDI scholarship will contribute only USD 2,318 as funds for research associated activities No other remunerations, benefits, compensation, stipend, fees or other costs will be supported through this funding.

5. Application for Tax Exemption

In order to qualify for tax exemption, the student must provide proof of enrollment in a recognized post-graduate degree program; and the student must remain in good standing as a registered student throughout the studentship period.

IDI will assist a qualifying student to apply for a formal, written tax exemption from the Uganda Revenue Authority (URA). The student's stipend from IDI, specified above, will be subject to **graduated PAYE tax only** until a written tax exemption is received from URA. IDI does not guarantee or represent that any application for tax exemption will be successful. The student will be solely responsible for any other tax or assessments on financial support received from IDI if any.

The student acknowledges that any breach of this covenant will immediately void any tax exemption received pursuant to this agreement and, upon learning of any breach of this covenant; IDI will immediately resume deducting and remitting all applicable income taxes.

5. Relationship with IDI

In achieving the aims of Section 1, above, the student will not become or act as an employee or agent of IDI. The student will not participate in IDI employee benefit plans nor receive any other compensation from IDI.

6. Term

The term of this Agreement shall be from **1st December 2019 to 31st May 2020.**

7. Termination

IDI and the student shall have the right to terminate this Agreement with thirty (30) days prior written notice for any reason. In the event of termination, IDI shall provide the normal stipend for the notice period and will reimburse the proportion of paid tuition up to the proportion that the completed duration of the agreement is to one year.

IDI shall terminate this contract if the student fails to notify it about any extra funding received for tuition.

8. Entire Agreement

This Agreement contains the entire understanding between the parties and super-cedes all prior agreements and understandings between the parties. No modifications or amendment to this Agreement shall be effective unless in writing and signed by each of the parties.

9. Choice of Law

This Agreement shall be governed by and construed and interpreted in accordance with the laws of the Republic of Uganda.

Student

By: ____________________

Ignatius Sentenza

College of Health Sciences

Makerere University

Mulago Hospital Complex

Date: 02/03/2020

Infectious Diseases Institute

By: ____________________

pp. Susan Lamunu Shereni
Head - Finance & Administration
Infectious Diseases Institute
College of Health Sciences
Makerere University
Mulago Hospital Complex
E-mail: slamunu@idi.co.ug

Date: 31/1/2020
