# Supplementary figures and images for: Virological non-suppression among adult males attending HIV care services in the fishing communities in Bulisa district, Uganda
Source: PLoS One. 2023 Oct 19;18(10):e0293057. doi: 10.1371/journal.pone.0293057 (PMC10586650; doi:10.1371/journal.pone.0293057)

[illegible]

## LOCATION OF BULISA DISTRICT IN UGANDA

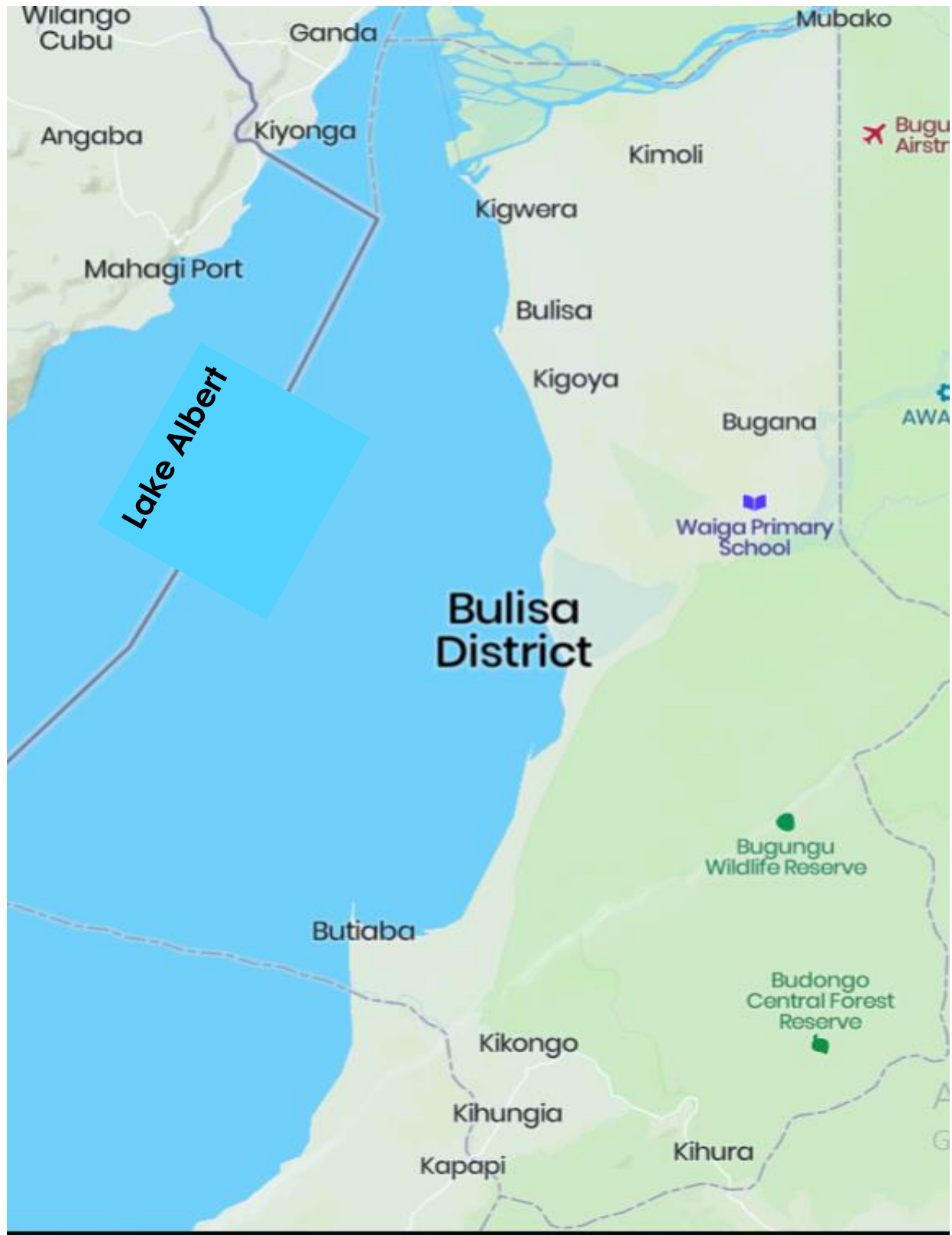

Supplement: S2 File — (PDF) [file pone.0293057.s002.pdf]
