# Supplementary material for: Virological non-suppression among adult males attending HIV care services in the fishing communities in Bulisa district, Uganda
Source: PLoS One. 2023 Oct 19;18(10):e0293057. doi: 10.1371/journal.pone.0293057 (PMC10586650; doi:10.1371/journal.pone.0293057)
Supplement: S4 File — (PDF) [file pone.0293057.s004.pdf]

CODE	
**INDIVIDUAL FACTORS**	
	**Current viral load**
0	Suppressed
1	Non supressed
	**Immediate previous viral load**
0	Suppressed
1	Non suppressed
	**Latest CD4**
0	Less than 200
1	200-500
2	Greater than 500
	**Diagnosed with TB in the last one year**
0	No
1	Yes
	**Current ART regimen**
0	TDF/3TC/EFV
1	TDF/3TC/DTG
2	TDF/3TC/ATV/r
3	AZT/3TC/NVP
4	Other second line
5	Other first line
	**Line of current regimen**
0	First
1	Second/ third
	**Duration on ART**
0	Less than 24 months
1	24-50 months
2	Greater than 50 months
	**Frequency of current ART regimen**
0	Once daily
1	Twice daily
	**Ever changed regimen from baseline**
0	yes
1	No
	**Body mass index**
0	Less than 18.5
1	18.5 to 24.9
2	Greater than or equal to 25.0
	**Mid upper arm circumference**
0	Green
1	Yellow/ red
	**Baseline CD4**
0	Less than 200
1	200-500
2	Greater than 500
	**Documented adherence level**
0	Good
1	Fair
2	Poor
	**Clinical Stage**
0	I
1	II
2	III and IV
	**More than 7 days appointment interruption in last 12 months**
0	0
1	1 to 3
2	More than 3
	**Side effects**

0	No
1	yes
	**Baseline ART**
0	AZT/3TC/NVP
1	AZT/3TC/EFV
2	TDF/3TC/EFV
3	TDF/3TC/DTG
4	Other regimens
	**Frequency of baseline regimen**
0	Once daily
1	Twice daily
	**Age category**
0	Greater than 50 years
1	26 to 50
2	15 to 25
	**Marital status**
0	Single
1	Divorced
2	Married
3	Cohabiting
4	Widowed
	**Ability to read and write**
0	No
1	Yes
	**Highest level of education**
0	No schooling
1	Completed primary
2	Completed secondary
3	Some Primary
4	Some secondary
	**Occupation**
0	Others jobs
1	Fishing / trading in fish
2	Not employed
	**Average income**
0	> UGX 20,000
1	< UGX 10,000
2	UGX 10,000 – 20,000
	**Religion**
0	Catholic
1	Born again
2	Muslim
3	Others
4	Protestant
	**Time to facility**
0	Less than 1 hour
1	1-2 hours
2	2 hours
	**Transport costs**
0	2000-5000
1	5000-10,000
2	Greater than 10,0000
	**Missed appointment because of transport**
0	No
1	Yes
	**Meals per day**
0	1 meal
1	2-3 meals

2	More than 3 meals
	**Missed ART because of food**
0	No
1	Yes
	**No of sexual partners**
0	None
1	Only one
2	More than one
	**Condom use**
0	Never
1	Once in a while
2	Every time
	**Partner HIV status**
0	Don’t know
1	Negative
2	Positive
	**Partner on ART**
0	No
1	Yes
	**Partner getting ART from same facility**
0	No
1	Yes
	**Knowledge of partner suppression**
0	Suppressed
1	Non suppressed
2	Don’t know
	**Missed taking ART in last 12 months**
0	No
1	yes
	**Reason for missing ART in last 12 months**
0	Transport / travel
1	Forgot
2	Was too sick
3	Lack of food
4	The work I do
5	Other reasons
	**Treatment supporter**
0	Don’t have
1	Work mate
2	Wife
3	Any other family member
	**Suffered a stressful life event following HIV diagnosis**
0	No
1	Yes
	**HIV disclosure**
0	No one
1	Any other family member
2	Work mate/ friend/neighbour
3	Wife
	**Frequency of spending nights outside home**
0	Never
1	Once a week
2	2-3 times a week
3	More than 3 times a week
	**Awareness of dangers of viral load**
0	No
1	Yes
	**Smoking behaviour**

0	Never
1	Used to some but quit
2	Tried once and stopped
3	Still smoke up to now
	**Inquisitive about vial load status**
0	No
1	Yes
	**Afraid about disclosing HIV status**
0	No
1	Yes
	**Health status after starting ART**
0	About the same
1	Can't tell
2	Getting worse
3	Improved
	**Missed clinic appointment in last 6 months**
0	No
1	Yes
	**Reason for missing appointment**
0	Forgot
1	Engaged in work
2	Feeling sick/ side effects
3	Travelled/ transport
	**Use of traditional medicines**
0	No
1	Yes
	**Lost interest in pleasurable activities**
0	No
1	Yes
	**Safety perception of HIV related drugs**
0	Have no problem
1	Have some problems
2	Have many problems
	**Hazardous use of alcohol**
0	Non-hazardous use
1	Hazardous use
	**Frequency of cross over to Congo in a year**
0	Never
1	Once or twice
2	More than twice
	**Frequency of moving between landing sites in a year**
0	More than twice
1	Once or twice
2	Never
	**Duration spend outside usual workplace due to mobility**
0	Less than 1 month
1	1 to 2 months
2	Three or more months
	**ARVs and stability at work**
0	No disruption
1	Disruption
	**Permanent resident of Bulisa**
0	No
1	Yes
	**Nationality**
0	Non Ugandan
1	Ugandan

	**Missed ARVs because of work**
0	No
1	Yes
HEALTH FACILITY FACTORS	
	**Participant's facility for treatment**
0	Facility at HCII level
1	Facility at HC III level
2	Facility at HC IV level
3	Facility at Hospital level
	**Taught about importance of viral load suppression**
0	No
1	Yes
	**Belonging to a treatment support group**
0	Yes
1	No
	**Ever missed appointment due to facility set up/ hygiene**
0	No
1	Yes
	**Assessment of attention given to concern expression**
0	Very good
1	Fair
2	Good
	**Engaged in selecting treatment options**
0	No
1	Yes
	**Experience when starting ART**
0	forced
1	Given time to make a decision
	**Assessment of confidentiality at facility**
0	No confidentiality
1	Some confidentiality
2	Maximum confidentiality
	**Waiting time at facility**
0	Very short
1	Short
2	long
3	Very long
	**Quality of health education talks**
0	Do not happen at all
1	Less interactive and rushed
2	Interactive
	**Quality of counselling sessions**
0	Given enough time
1	rushed
	**Satisfaction of answering HIV related questions**
0	Very satisfied
1	Satisfied

2	Not very satisfied
	**Ever missed drugs because of drug stock outs**
0	No
1	Yes
	**Quality of clinical appointments and work**
0	Long enough
1	They are just fine
2	Too short
	**Perceived competency of health workers**
0	All are knowledgeable
1	Some lack knowledge
2	All lack knowledge
	**HIV services extended near work**
0	Yes
1	No
	**Made to understand my viral load**
0	No
1	Yes
	**Viral load turnaround time**
0	Can’t tell
1	1 to 2 months
2	More than 2 months
3	Less than 1 month
	**Health work gentleness**
0	Never shout
1	Sometimes shout at me
2	Always shout at me
	**Pill balance assessment**
0	Every time
1	Some times
2	Never been asked
	**Counselling session**
0	Given individually
1	Given in a group
2	Both individual and group
3	Never been given
