# Supplementary material for: Virological non-suppression among adult males attending HIV care services in the fishing communities in Bulisa district, Uganda
Source: PLoS One. 2023 Oct 19;18(10):e0293057. doi: 10.1371/journal.pone.0293057 (PMC10586650; doi:10.1371/journal.pone.0293057)
Supplement: S5 File — (PDF) [file pone.0293057.s005.pdf]

CONSENT FORM – ENGLISH

This informed consent form is for HIV positive adult males (15 years or older) with the most recent viral load done within 12 months before data collection

Name of Principal Investigator: Senteza Ignatius

Name of institution: Makerere University School of Public Health

Name of Sponsor: Senteza Ignatius

Name of Proposal: Factors associated with virological non-suppression among HIV positive adult males (15 years or older) in the fishing communities of Bulisa district.

PART I:

Introduction

Good morning/afternoon, I am Senteza Ignatius and I am doing a study on the factors associated with virological non-suppression among HIV positive adult males (15 years or older) in the fishing communities of Bulisa district.

I am going to give you information and invite you to participate in this study. However, before you decide, you can talk to anyone you feel comfortable with. There may be some words that you do not understand. Please ask me to stop as we go through the information and I will take time to explain. If you have questions later, you can still ask them of me or the health workers to provide the appropriate answers.

Purpose

Bulisa district has a problem of HIV virological non-suppression among adult males (15 years) and above. HIV virological non-suppression is associated with increased chances of HIV transmission. Since fishing communities have been reported to have a high prevalence of HIV, promoting virological suppression is a good way to cut on new HIV infections. The study aims to determine the factors associated with virological non-suppression among HIV positive adult males (15 years or older) in the fishing communities of Bulisa district. This research is being conducted for dissertation/academic purposes only

Participant selection

HIV virological non-suppression is a big problem among adult males (15 years or older) in Bulisa district. We believe that you are a resourceful person in addressing this problem. We are inviting you to take part in this study because it is important that we get to know the factors associated with virological non-suppression among adult males (15 years or older) in the fishing communities of Bulisa district.

Voluntary Participation

Your decision to participate in this study is voluntary. It is your choice to participate or not. If you choose not to consent, all the services you receive from the health facility and other programs will continue and nothing will change.

Procedures

There is not going to be any procedure that will cause pain or discomfort to you. You will be required to only respond to questions.

Duration
The estimated time to complete this study is approximately 20 minutes. Once we complete filling this form (questionnaire) with you, may leave the study at any time. If you decide to stop participating, there will be no penalty to you

Risks, Discomforts and Benefits
Your participation in this study does not involve any physical or emotional risk or damage to you. You will receive no immediate benefits from participating in this research but your participation will enable us to determine the factors associated with virological non-suppression among HIV positive adult males (15 years or older) in the fishing communities of Bulisa district.

Confidentiality
The information that we collect from this study will be kept confidential. Personal identifying information (i.e. names) about you will not be captured but no one but the researcher will access the other information that will be collected from the study. Any information about you will have a number on it instead of your names. Only the principal investigator and the researchers will know whose number it is and we will lock that information up in a safe place that cannot be easily accessed by any one. Your name or other identifying information will not be used in our reports or published papers.

Sharing of the results
The knowledge and understanding that we get from this study will be shared with the school of public health, Makerere university, Bulisa district DHOs office and other stakeholders. However, confidential information will not be shared.

Who to Contact
If you have any questions, you may ask them now or later, even after the study has started. If you wish to ask questions later, you may contact the following: Dr Senteza Ignatius 0773027744. This proposal has also been reviewed and approved by Makerere University school of public Health Review Board, which is a committee whose task it is to make sure that study participant is protected from harm.

Participant Rights
Participation in this study is voluntary. You are free to leave the study at any time without any penalty.
Your decision not to participate is no way influences any future participation in the research.
Do you have any questions about the above information? Do you wish to participate in this study? Yes
No ☐ ☐

PART II: Certificate of Consent

Signature of participant ____________________Date __________________Day/month/year

Statement by the researcher/person taking consent __________________________________

Name of Researcher ____

Signature of Researcher/person taking consent: ___________________Date _____________

ASSENT FORM – ENGLISH

This informed assent form is for HIV positive adult males (15 years to less than 18 years) with the most recent viral load done within 12 months prior to the date collection

Name of Principal Investigator: Senteza Ignatius

Name of institution: Makerere University School of Public Health

Name of Sponsor: Senteza Ignatius

Name of Proposal: Factors associated with virological non-suppression among HIV positive adult males (15 years or older) in the fishing communities of Bulisa district.

PART I:

Introduction

Good morning/afternoon, I am Senteza Ignatius and I am doing a study on the factors associated with virological non-suppression among HIV positive adult males (15 years or older) in the fishing communities of Bulisa district.

I am going to give you information and invite you to participate in this study. However, before you decide, you can talk to anyone you feel comfortable with. There may be some words that you do not understand. Please ask me to stop as we go through the information and I will take time to explain. If you have questions later, you can still ask them of me or the health workers to provide the appropriate answers.

Purpose

Bulisa district has a problem of HIV virological non-suppression among adult males (15 years) and above. HIV virological non-suppression is associated with increased chances of HIV transmission. Since fishing communities have been reported to have a high prevalence of HIV, promoting virological suppression is a good way to cut on new HIV infections. The study aims to determine the factors associated with virological non-suppression among HIV positive adult males (15 years or older) in the fishing communities of Bulisa district. This research is being conducted for dissertation/academic purposes only

Participant selection

HIV virological non-suppression is a big problem among adult males (15 years or older) in Bulisa district. We believe that you are a resourceful person in addressing this problem. We are inviting you to take part in this study because it is important that we get to know the factors associated with virological non-suppression among adult males (15 years or older) in the fishing communities of Bulisa district.

Voluntary Participation

Your decision to participate in this study is voluntary. It is your choice to participate or not. If you choose not to consent, all the services you receive from the health facility and other programs will continue and nothing will change.

Procedures

There is not going to be any procedure that will cause pain or discomfort to you. You will be required to only respond to questions.

Duration

The estimated time to complete this study is approximately 20 minutes. Once we complete filling this form (questionnaire) with you, may leave the study at any time. If you decide to stop participating, there will be no penalty to you

Risks, Discomforts and Benefits

Your participation in this study does not involve any physical or emotional risk or damage to you. You will receive no immediate benefits from participating in this research but your participation will enable us to determine the factors associated with virological non-suppression among HIV positive adult males (15 years or older) in the fishing communities of Bulisa district.

Confidentiality

The information that we collect from this study will be kept confidential. Personal identifying information (i.e. names) about you will not be captured but no one but the researcher will access the other information that will be collected from the study. Any information about you will have a number on it instead of your names. Only the principal investigator and the researchers will know whose number it is and we will lock that information up in a safe place that cannot be easily accessed by anyone. Your name or other identifying information will not be used in our reports or published papers.

Sharing of the results

The knowledge and understanding that we get from this study will be shared with the school of public health, Makerere university, Bulisa district DHOs office and other stakeholders. However, confidential information will not be shared.

Who to Contact

If you have any questions, you may ask them now or later, even after the study has started. If you wish to ask questions later, you may contact the following: Dr Senteza Ignatius 0773027744. This proposal has also been reviewed and approved by Makerere University school of public Health Review Board, which is a committee whose task it is to make sure that study participant is protected from harm.

Participant Rights

Participation in this study is voluntary. You are free to leave the study at any time without any penalty. Your decision not to participate in no way influences any future participation in the research.

Do you have any questions about the above information? Do you wish to participate in this study? Yes
No ☐ ☐

PART II: Certificate of Consent

Signature of guardian for the participant ________________Date ______________Day/month/year

Statement by the researcher/person taking consent __________________________________

Name of Researcher ____

Signature of Researcher/person taking consent: ___________________Date _____________
