# Supplementary material for: Virological non-suppression among adult males attending HIV care services in the fishing communities in Bulisa district, Uganda
Source: PLoS One. 2023 Oct 19;18(10):e0293057. doi: 10.1371/journal.pone.0293057 (PMC10586650; doi:10.1371/journal.pone.0293057)
Supplement: S6 File — (PDF) [file pone.0293057.s006.pdf]

CONSENT FORM – RUGUNGU VERSION

LUPAPURA LUGABA RUKUSA

Lulu Luli lupapura lukugaba rukusa mu batulu bahandu bali na kasiisa ka munywereeru ba myaka kumi na mitaanu rundi kukiraho bapimiirwe bunene bwa kasiisa ka munywereeru mu mbirir myabu mu keire ka mweri ikumi na mibiri hehi na kusorooza aga makuru mu bantu.

Mabara ga Musaki waamakuru Senteza Ignatius mabara geisomero Makerere University school og public health.

Akutamwo nsimbi/ musagiki senteza Ignatius mabara ga kitekerezo nsonga zikukwatagana ne butakendera bwa kasiisa kamunywereru mubadulu ba myaka 15 rundi kukiraho mu mitanda ja nyamasaza gya Bulisa

NDIGIIRO/ NTANDIKO

Mwalere teetei/ musibiri teetei, mba senteza ndi mu kwekebeija nsonga zikwatagna na butakendera bwa kassisa ka munywereru mu badulu bahandu ba myaka ikumi na mitaanao mya buhandu na kukiraho (myaka 15) bali na kasiisa ka munywereru.

Nkugyenda kubaha makuru kandi mbeete tukoragane nanywe mu kwekebei ja kuku. Kyonkei mutakabbeeri kucwamu mu kusobora kubuulya mu mitanda mya nyamasaza gya bulisa munta yensei kgimuwona akusobora rundi ataali na kaku kense. Hasobora kubbaho bigambu bindi byensei bimutakwetegereza babasoboore. Musobora kunsaba nyeemeere niturabyanga mumeiso mu makuru gatutungiri kandi mutunge bwire waakusbasobora. Heinyuma waakabba na bibuulyo okusobora kubuulya gya rundi mukori wa heirwaru akuhe miramu gisemereeri kando gidoori.

KIGYENDERERWA

Nyamasaza gya Bulisa gila na kizibu Kya butakendera bwa kasiinsa kamunyereru mubaddulu bahandu ba myaka ikumi na mitaanu (myaka 15) na kugyenda mumeiso.

Kikikwatagana na migisa minene mya kutuura. Kizookirir nti mu mitanda/ mu balobi kutuura kwa kasiisa ka silimu kuli kwakyendi hoi.

Mulingo gumwei gwakukeehya kukwatwa kasiisa buhyaka. Kyegyendererwa kwa kuku kwekebeija kili kutunga nsonga zikuletereza ku Kya butakendera bwa kasiinsa kamunyereru kamunyereru mubaddulu bahandu ba myaka ikumi na mitaanu (myaka 15) na kugyenda mumeiso mu mitanda mya nyamasaza gya bulisa. Kutolererya kwabbeeriho inyuma na ripoota baagi hulikiirye.

KUKOMA BAKUKWATAHO

butakendera bwa kasiinsa kamunyereru kiki kiri kizibu Kyamaani hoi mu mubaddulu bahandu ba myaka ikumi na mitaanu (myaka 15) na kugyenda mumeiso mu mitanda mya nyamasaza gya Bulisa. Tukwiriza nti oli omwei mu bantu bamagezi kandi bakusoboro kumala kkiki kizibu. Tukukweta tubbe nawe mu kuku kwekebeija habwa kubba kili kya mugasu twege zizi nsoga zikukwatagana na kasiinsa kamunyereru kamunyereru mubaddulu bahandu ba myaka ikumi na mitaanu (myaka 15) na kugyenda mumeiso mu mitanda/ mu balobi mya nyamasaza gya Bulisa

KWETABA KUBBA KWEKEBEIJA

Gili ncwamu gyamu kwetaba natwe mu kuku kwekebeija kandi kili ha kwendya kwamu (okusasulwa kwahimusaara). Okwecwekamwo wankei kukora natwe nundu kusuula. Kyonkei kadi bwa kasuula kukora natwe bukoonyezi bwoic eeri notunga kuruga heirwaru bukugyenda mumeiso hataloho mpiduka.

MIGYENDERE

Hakubbaho kwahi migyendere mindi myensei mikusobora kukudoosyaho burumi rundi kwelaalikiira kundi kwensei. Okusabwa kwiramu bibi bibuulyo bisa.

BWIRE

Bwire bubaliirwe mu kuku kwekebeija ziri dakika makumi gabiri (dakiika 20). Waakamala kwiramu bibuulyo byona byo, okusobora kugyenda mu bwire bwamu bwensei. Ncwawo gyamu kulekaho kukora natwe gili kwahi na kifubiro kindi kyensei.

BIZIBU, KWERALIKIRIIRA NA BURUNGI

Kwetaba kwamu mu kuku kwekebeija hali mu kwahi kizibu kindi kyensei rundi kuhutaazibwa kundi kwensei. Oiza kutunga Birungi ha bwire bwo – bwo kuruga mu bakukwatwahoona mu kuku kusaa kwa makuru kyonkei kwetaba kwamu kukwiza kukoonyeera mutunga nsonga zikwatagna na butakendera bwa kassisa ka munywereru mubaddulu bahandu ba myaka ikumi na mitaanu (myaka 15) na kugyenda mumeiso mu mitanda/ mu balobi mya nyamasaza gya Bulisa.

KULINDA NSITA

Makura gatukutunga kuruga mu kuku kwekebeija ga kwiza kubiikwa mu nsita gya maani hoi. Makuru ga kukubazaho mka mabara gakwizakwahi kuhandiikwa,musaki wa makuru akwiza Kukoresya Suura. Kandi musaki akwiza kuhandiika makuru yandi gensei kuruha mu kuku kwekebeija.

Musaki muhandu na beira basa boobo bakwiza kwega zizyo suura kandi tukwiza kubiika gagwa makuru mu kiikaru kirungi hoi kandi kya nsita ha buli muntu yensei atakusobora kudwa.

Mabara rundi bikukubazaho bikwiza kwahi Kukoresebwa mu ripoota gyetu rundi Kubihulukya mu Mpapura zya Makuru.

KUBAGANA BITWAGIRYE/ BIRUGIRI MU KUSAKA

Magezi na kwetegereza kututungiri kurunga mu kuku kwekebeija kukwiza kubaganwa itendekero lya Makerere Yunivaasite lya bwami bwa bantu, muhandu wa bya bwomi mu Nyamasaza gya Bulisa na bakukwatwaho. Kyonkei makuru ga nsita gakwiza kwahi kubanga nwa/ kubazwaho.

WA KUDWEREERA

Waakabba na bibuulyo byensei okusobora kubulya hataati rundi bwire bundi/ heinyuma kadi kwekebeija kwakabba kutandikiri. Waakabba ocwirimu kubuulya heinyuma, okusobora kudwerera baba dakitaali Senteza Kusuura za simu zizi 0773027744. Kiki kitekerezo kyarabirwemo meiso kandi kiteebwaho Mukono ba kakurato keitendekero Mukono ba kakurato keitendekero lya Makerere Yunivasite kajunaanwa kwekebeija bya bwomi bwa bantu. Katebe Kasabirwe kuwomera kimwe nti bantu bakukwataho balindirwe kurungi kandi tibahutazibiirwe.

BUGABE BWA BAKUKWATWAHO

Kwetaba mu kwekebeija kuli kwa busa oli wa Bugabe kugyenda rundi kuleka kuku kwekebeija mu bwire bwamu bwennsei hataloho kitubiro kindi kyensei. Ncwamu gyamu kukora natwe gikwiza kwahi kuhambiriza kwetaba mu kuku kusaka kwa makuru.

Olin a kibuulyo kindi kyensei kiku kwatagana na makuru gali hakyendi?

Okwendya Kwetaba mu kuku kwekebeija? Kwokwo _____________ Kwahi ______________

KICHEKA KYA II

LUPAPURA LUGABA RUKUSA

Mukono gwa akukwatwaho _________________________ biro bya mweri __________________ kiro/ mweri/ mwaka ____________________________________

Bigambu bya musaki ____
____

Mabara ga musaki ____

Mukono gwa musaki _________________________________ Biro __________________________

ASSENT FORM – RUGUNGU VERSION

LUPAPURA LUGABA RUKUSA

Lulu Luli lupapura lukugaba rukusa mu batulu bahandu bali na kasiisa ka munywereeru ba myaka kumi na mitaanu kudwa hamyaka kuminamunaani bapimiirwe bunene bwa kasiisa ka munywereeru mu mbirir myabu mu keire ka mweri ikumi na mibiri hehi na kusorooza aga makuru mu bantu.

Mabara ga Musaki waamakuru Senteza Ignatius mabara geisomero Makerere University school og public health.

Akutamwo nsimbi/ musagiki senteza Ignatius mabara ga kitekerezo nsonga zikukwatagana ne butakendera bwa kasiisa kamunywereru mubadulu ba myaka 15 rundi kukiraho mu mitanda ja nyamasaza gya Bulisa

NDIGIIRO/ NTANDIKO

Mwalere teetei/ musibiri teetei, mba senteza ndi mu kwekebeija nsonga zikwatagna na butakendera bwa kassisa ka munywereru mu badulu bahandu ba myaka ikumi na mitaanao mya buhandu na kukiraho (myaka 15) bali na kasiisa ka munywereru.

Nkugyenda kubaha makuru kandi mbeete tukoragane nanywe mu kwekebei ja kuku. Kyonkei mutakabbeeri kucwamu mu kusobora kubuulya mu mitanda mya nyamasaza gya bulisa munta yensei kgimuwona akusobora rundi ataali na kaku kense. Hasobora kubbaho bigambu bindi byensei bimutakwetegereza babasoboore. Musobora kunsaba nyeemeere niturabyanga mumeiso mu makuru gatutungiri kandi mutunge bwire waakusbasobora. Heinyuma waakabba na bibuulyo okusobora kubuulya gya rundi mukori wa heirwaru akuhe miramu gisemereeri kando gidoori.

KIGYENDERERWA

Nyamasaza gya Bulisa gila na kizibu Kya butakendera bwa kasiinsa kamunyereru mubaddulu bahandu ba myaka ikumi na mitaanu (myaka 15) na kugyenda mumeiso.

Kikikwatagana na migisa minene mya kutuura. Kizookirir nti mu mitanda/ mu balobi kutuura kwa kasiisa ka silimu kuli kwakyendi hoi.

Mulingo gumwei gwakukeehya kukwatwa kasiisa buhyaka. Kyegyendererwa kwa kuku kwekebeija kili kutunga nsonga zikuletereza ku Kya butakendera bwa kasiinsa kamunyereru kamunyereru mubaddulu bahandu ba myaka ikumi na mitaanu (myaka 15) na kugyenda mumeiso mu mitanda mya nyamasaza gya bulisa. Kutolererya kwabbeeriho inyuma na ripoota baagi hulikiirye.

KUKOMA BAKUKWATAHO

butakendera bwa kasiinsa kamunyereru kiki kiri kizibu Kyamaani hoi mu mubaddulu bahandu ba myaka ikumi na mitaanu (myaka 15) na kugyenda mumeiso mu mitanda mya nyamasaza gya Bulisa. Tukwiriza nti oli omwei mu bantu bamagezi kandi bakusoboro kumala kkiki kizibu. Tukukweta tubbe nawe mu kuku kwekebeija habwa kubba kili kya mugasu twege zizi nsoga zikukwatagana na kasiinsa kamunyereru kamunyereru mubaddulu bahandu ba myaka ikumi na mitaanu (myaka 15) na kugyenda mumeiso mu mitanda/ mu balobi mya nyamasaza gya Bulisa

KWETABA KUBBA KWEKEBEIJA

Gili ncwamu gyamu kwetaba natwe mu kuku kwekebeija kandi kili ha kwendya kwamu (okusasulwa kwahimusaara). Okwecwekamwo wankei kukora natwe nundu kusuula. Kyonkei kadi bwa kasuula kukora natwe bukoonyezi bwoic eeri notunga kuruga heirwaru bukugyenda mumeiso hataloho mpiduka.

MIGYENDERE

Hakubbaho kwahi migyendere mindi myensei mikusobora kukudoosyaho burumi rundi kwelaalikiira kundi kwensei. Okusabwa kwiramu bibi bibuulyo bisa.

BWIRE

Bwire bubaliirwe mu kuku kwekebeija ziri dakika makumi gabiri (dakiika 20). Waakamala kwiramu bibuulyo byona byo, okusobora kugyenda mu bwire bwamu bwensei. Ncwawo gyamu kulekaho kukora natwe gili kwahi na kifubiro kindi kyensei.

BIZIBU, KWERALIKIRIIRA NA BURUNGI

Kwetaba kwamu mu kuku kwekebeija hali mu kwahi kizibu kindi kyensei rundi kuhutaazibwa kundi kwensei. Oiza kutunga Birungi ha bwire bwo – bwo kuruga mu bakukwatwahoona mu kuku kusaa kwa makuru kyonkei kwetaba kwamu kukwiza kukoonyeera mutunga nsonga zikwatagna na butakendera bwa kassisa ka munywereru mubaddulu bahandu ba myaka ikumi na mitaanu (myaka 15) na kugyenda mumeiso mu mitanda/ mu balobi mya nyamasaza gya Bulisa.

KULINDA NSITA

Makura gatukutunga kuruga mu kuku kwekebeija ga kwiza kubiikwa mu nsita gya maani hoi. Makuru ga kukubazaho mka mabara gakwizakwahi kuhandiikwa,musaki wa makuru akwiza Kukoresya Suura. Kandi musaki akwiza kuhandiika makuru yandi gensei kuruha mu kuku kwekebeija.

Musaki muhandu na beira basa boobo bakwiza kwega zizyo suura kandi tukwiza kubiika gagwa makuru mu kiikaru kirungi hoi kandi kya nsita ha buli muntu yensei atakusobora kudwa.

Mabara rundi bikukubazaho bikwiza kwahi Kukoresebwa mu ripoota gyetu rundi Kubihulukya mu Mpapura zya Makuru.

KUBAGANA BITWAGIRYE/ BIRUGIRI MU KUSAKA

Magezi na kwetegereza kututungiri kurunga mu kuku kwekebeija kukwiza kubaganwa itendekero lya Makerere Yunivaasite lya bwami bwa bantu, muhandu wa bya bwomi mu Nyamasaza gya Bulisa na bakukwatwaho. Kyonkei makuru ga nsita gakwiza kwahi kubanga nwa/ kubazwaho.

WA KUDWEREERA

Waakabba na bibuulyo byensei okusobora kubulya hataati rundi bwire bundi/ heinyuma kadi kwekebeija kwakabba kutandikiri. Waakabba ocwirimu kubuulya heinyuma, okusobora kudwerera baba dakitaali Senteza Kusuura za simu zizi 0773027744. Kiki kitekerezo kyarabirwemo meiso kandi kiteebwaho Mukono ba kakurato keitendekero Mukono ba kakurato keitendekero lya Makerere Yunivasite kajunaanwa kwekebeija bya bwomi bwa bantu. Katebe Kasabirwe kuwomera kimwe nti bantu bakukwataho balindirwe kurungi kandi tibahutazibiirwe.

BUGABE BWA BAKUKWATWAHO

Kwetaba mu kwekebeija kuli kwa busa oli wa Bugabe kugyenda rundi kuleka kuku kwekebeija mu bwire bwamu bwennsei hataloho kitubiro kindi kyensei. Ncwamu gyamu kukora natwe gikwiza kwahi kuhambiriza kwetaba mu kuku kusaka kwa makuru.

Olin a kibuulyo kindi kyensei kiku kwatagana na makuru gali hakyendi?

Okwendya Kwetaba mu kuku kwekebeija? Kwokwo ______________ Kwahi _______________

KICHEKA KYA II

LUPAPURA LUGABA RUKUSA

Mukono gwa akukwatwaho ___________________________ biro bya mweri ___________________ kiro/ mweri/ mwaka ______________________________________

Bigambu bya musaki ____
____

Mabara ga musaki ____

Mukono gwa musaki ___________________________________ Biro ____________________________
