# Supplementary material for: Virological non-suppression among adult males attending HIV care services in the fishing communities in Bulisa district, Uganda
Source: PLoS One. 2023 Oct 19;18(10):e0293057. doi: 10.1371/journal.pone.0293057 (PMC10586650; doi:10.1371/journal.pone.0293057)
Supplement: S7 File — (PDF) [file pone.0293057.s007.pdf]

PATIENT'S QUESTIONNAIRE (ENGLISH VERSION)

UNIQUE IDENTIFIER ☐

This Questionnaire is to be applied to only male adults (15 years) residing in the fishing communities of Bulisa district on ART for atleast 6 months. The patient must be seeking care at one of the health facilities in Bulisa district

PART ONE: DEMOGRAPHIC CHARACTERISTICS

1. **What is your current age** ____
2. **What is your current marital status?** ____

   a) Married officially **b)** Single **c)** Divorced **d)** cohabiting **e)** widowed **f)** others (specify) _____

3. **Are you able to read and write?**

   a) Yes **b)** No

4. **What is your highest education level achieved?**

   a) No schooling **b)** some primary **c)** completed primary **d)** some secondary **e)** completed secondary **f)** some university **g)** completed secondary

5. **Current employment status**

   a) Not employed **b)** fishing **c)** trading in fish **d)** casual labourer e**)** others (specify)____

6. **What is your daily income on average?**

   a) Less than Ush.10, 000 **b)** between Ush.10, 000 and 20,000 **c)** more than Ush. 20,000

7. **What is your religion?**

   a) Catholic **b)** Muslim **c)** Protestant **d)** Other (specify) _______________

<u>PART TWO: INDIVIDUAL FACTORS ASSOCIATED WITH VIROLOGICAL NON-SUPPRESSION AMONG ADULT MALES (15 YEARS OR OLDER)</u>

8. **How much time does it take you to reach the facility?**

   a) Less than 1 hour **b)** 1- 2 hours **c)** more than 2 hours

9. **How much money in form of transport do you need to reach this health facility?**

   a) Less than Ush.2000 **b)** Ush. 2000 – 5000 **c)** 5000 – 10,000 **d)** more than 10,000

10. **Have you ever missed coming to the facility because you never had transport?**

    a) Yes **b)** No

11. **On average how many meals do you take in a day?**

    a) 1 meal **b)** 2-3 meals **c)** more than 3 meals

12. **Have you ever missed taking your ARVs because of no food?**

    a) Yes **b)** no

13. **How many sexual partners have you had in the last 12 months?**

    a) Only one **b)** 2-3 **c)** More than 3

14. **How often was a condom used during sexual intercourse in the last 12 months?**

    a) Every time **b)** once in a while **c)** never

15. **What is the HIV status of your spouse?**

    a) positive **b)** Negative **c)** don't know

16. **If the spouse is positive, is she on ART?**

    a) Yes **b)** No

17. **If the spouse is positive, are you getting HIV drugs from the same facility?**

    a) Yes **b)** no

18. **If the spouse is positive, what is the latest virological suppression status**

    a) Suppressed **b)** non suppressed **c)** don't know

19. **Have you missed taking your ARVs in the last 12 months?**

    a) Yes **b)** No

20. If you have missed some doses, what made you miss drugs in the most recent episode

    a) The work I do **b)** I forgot **c)** I was too sick **d)** No drugs at the facility

    e) Side effects **f)** others specify ________________________________

21. **Who is your treatment supporter?**

    a) I don't have b) work mate c) wife d) child e) parent f) other (specify) ___________________________________

22. **Have you experienced any form of bad outcome because of having HIV?**

    a) Yes **b)** No

23. **Who have you disclosed your HIV status to**

    a) Wife **b)** children **c)** workmates **d)** any other family member **e)** no one

24. **How often do you sleep outside your home in a week?**

    a) Never **b)** Only once a week **c)** 2-3 times a week **d)** more than 3 times a week.

25. **Do you belong to any treatment support group?**

    a) Yes **b)** No

26. **Are you aware a non-suppressed viral load can mean danger to your health?**

    a) Yes **b)** No

27. **Which of the following describes your smoking behaviour?**

a) Never smoked **b)** Tried once or twice **c)** Used to smoke but have quitted for months **d)** still smoke now

28. **Have you ever been taught about the importance of having a suppressed viral load?**

    a) Yes **b)** No

29. **On the last visit, did you ask the health worker your most up-to-date viral load result?**

    a) Yes **b)** no

30. **Are you afraid of people knowing that you are HIV positive?**

    a) Yes **b)** No

31. **Comparing your health before and starting ART, how would assess your health?**

    a) Improved **b)** about the same **c)** getting worse **d)** can't tell

32. **Have you missed a clinic appointment in the last 6 months?**

    a) Yes **b)** no

33. **What are some of the reasons that caused your missed appointment?**

    a) Got so engaged with work **b)** transport challenges **c)** forgot **d)** clinic staff disrespectful **e)** other specify

34. **Have you used traditional medicines to manage some of the medical problems in the last year?**

    a) Yes, **b)** No.

35. **Are there instances you have experienced both of the following in the past 6 months? Lost interest in pleasurable activities and felt hopeless**

    a) Yes **b)** No

36. **From your assessment, the treatment that you are taking now has**

    a) So many problems **b)** has some problems **c)** has no problem

37. **How often do you have a drink containing alcohol?**

    a) Never b) monthly or less c) 2-4 times a month d) 2-3 times a week e) 4 or more times a week

38. **How many standard drinks containing alcohol do you have on a typical day?**

    a) 1 or 2 b) 3 or 4 c) 5or 6 d) 7 to 9 e) 10 or more

39. **How often do you have six or more drinks on one occasion?**

    a) Never b) less than monthly c) monthly d) weekly e) daily or almost daily

40. **In a year, how often do you cross over to the Congo side of lake Albert**

    a) Never b) 1-2 times c) three or more times

41. **How often do you move from one landing site to another in a year?**

    a. Never b) 1-2 times c) three or more times

42. **In case you move, how long do you stay outside your usual working place?**

    a) Less than 1 month b) 1-2 months c) three or more times

43. **How would you grade the disruption caused by ARVs towards your work?**

a) No disruption b) some disruption c) very disruptive

44. **Are you a permanent resident of Bulisa district or you are a visitor?**
    a) Yes b) No.
45. **What is your nationality?**
    a) Ugandan b) Congolese c) others (specify) ___________________________
46. **Have you missed taking your drugs because of the nature of your work**
    a) Yes b) No

PART 3: HEALTH FACILITY FACTORS ASSOCIATED WITH VIROLOGICAL NON-SUPPRESSION AMONG ADULT MALES (15 YEARS OR OLDER)

47. **You have ever failed to come to the facility because the facility including toilets were dirty/ smelling bad**
    a) Yes b) No
48. **How do you gauge the facility in giving you enough time to express your concerns?**
    a) Very good b) fair c) poor.
49. **Have you been consulted about your preferences over alternative treatment options?**
    a) Yes b) No
50. **What do you have to say about your experience when you were starting ARVs?**
    a) Forced to start b) was given time to make decision c) others specify ________
51. **How would you score the facility design in considering the confidentiality of your HIV status?**
    a) No confidentiality b) some confidentiality c) total confidentiality
52. **How would you describe the time you take at the facility?**
    a) Very long b) long c) short d) very short
53. **Health education talks at this facility**
    a) Are interactive b) less interactive and rushed c) do not happen at all.
54. **About the quality of counselling sessions at this facility**
    a) Given enough time to understand the actual problem b,) rushed
55. **Satisfaction with how health workers have answered your questions about HIV**
    a) Very satisfied b) satisfied c) Not very satisfied
56. **You have ever been told to go back home because there are no drugs**
    a) Yes b) No
57. **About appointments given and the nature of my work**
    a) Too short for my type of work b) They are okay c) they are long enough to give me time to work
58. **Your feel about health workers that normally attend to me**
    a) All are knowledgeable b) some lack knowledge c) all don't know enough about HIV
59. **HIV treatment services have been extended nearer to your home or place of work**
    a) Yes b) No
60. **I have been made to understand my viral load result by my doctor**
    a) Yes b) No
61. **Period of receiving results for my viral load test after bleeding**
    a) Less than 1 month b) 1-2 months c) more than 2 months
62. **About gentleness of health workers**
    a) Always shout at me b) sometimes shout c) never shout at me

63. **About asking you about the pill balances in the last year**

a) Every time b) sometimes c) never been asked

64. **In the last counselling session I had**

a) Was given me to alone b) it was given in a group alone c) both individual and group.
