# Supplementary material for: Virological non-suppression among adult males attending HIV care services in the fishing communities in Bulisa district, Uganda
Source: PLoS One. 2023 Oct 19;18(10):e0293057. doi: 10.1371/journal.pone.0293057 (PMC10586650; doi:10.1371/journal.pone.0293057)
Supplement: S8 File — (PDF) [file pone.0293057.s008.pdf]

APPENDIX VI: PATIENT'S QUESTIONNAIRE (LUGUNGU VERSION)

UNIQUE IDENTIFIER [ ]

KICHWEKA KYAKUBANZA: BIKUKUBAZAHO

1. **Olinamyaka minghai..**
2. **Olimufumbo?**
   a) ndi wampita b) Ndikwahi na mukali c) Twahukeini d) ndinamukunzi e) ndimuferwa f) bindi..................................
3. **Okusobora kuhandika na kusoma?**
   a) ee b) kwahi
4. **Wasomiri kudwa hanya?**
   a) nyasomiri kwahi b) nyasomiri ho pulimare c) nyamari pulimare d) nyasomiriho siniya e) nyamari siniya f) nyasomiriho univasite g) nyamari univasite
5. **Olinamulimo kyani hatati?**
   a) mulimo gwahai b) kuloba c) ntuda nsu e) nyekolesya mbe f) bindi..........
6. **Oingirya zingahai bulikiro mukugeryagerya?**
   a) hansi wa silingi 10,000 b) hagati wa silingi 10,000 na 20,000 c) hakyendi wa silingi 20,000
7. **Osoma dini kyani**
   a) mukatuliki b) musiramu c) mukristayo d) gindi.........

KICHWEKA KYAKABIRI: NSONGA KYANI ZIKULETERERYA MUNTU KUBA NA KASINSA KATAKWIRA HANSI MU BADULU BAHANDU (MYAKA 15 NA KUKIRAHO)

8. **Kikutwalira bwire kyani kudwa hairwaru**
   a) hansi wa saha 1 b) hagati wa saha 1 na 2 c) kukiramu saha 2
9. **kikwetagisya sente zinghai kudwa hairwaru**
   a) hansi wa silingi 2000 b) hagati wa silingi 2000 na 5000 c) hagati wa silingi 5000 na 10,000 d) kukiramu silingi 10,000
10. **Wosiriho kweza hairwaru habwa kulemwa sente za ndibata?**
    a) ee b) kwahi
11. **Mukugeryagerya odya mirundi minghai mukiro?**
    a) hansi wa murundi gumwei b) hagati wa mirundi 2 na 3 c) kukira mirundi 3
12. **Wosiriho kumera mibazi myakasinsa ka munywerero habwakuba walingikwahi na kyakudya?**
    a) ee b) kwahi
13. **Otereini nabantu banghai mu myeri ikumi na mibiri mi rabiriho**
    a) omwei musa b) babiri rundi basatu c) kukiryamu ha basatu
14. **Kapiira wakakoreserye mirundi minghai nolimukikorwa kyabakwendyangana mu myeri ikumi na mibiri mi rabiriho**
    a) bulikeire b) murundi gumwe na gumwe c) Nyakakoreserye kwahi
15. **Mwirawamu yemeri teete mukigambo kikwatageini na kasinsa ka munwereru**
    a) alinako b) kalikahi c) nyegirikwahi

16. **Mukali wamu yakaba nali na kasinsa ka munwereru, ali ha mibazii?**

a) ee b) kwahi

17. **Mukali wamu yakaba nali na kasinsa ka munwereru, mibazii mumitunga kuruga hairwaru limwei?**

a) ee b) kwahi

18. **Mukali wamu yakaba nali na kasinsa ka munwereru, musai gwamwe gwalingi teete mukusembayo hoi**

a) kasinsa kalingi hansi b) kasinsa kalingi kwakyendi c) nyegirikwahi

19. **Wosiriho kumera mibazi myamu mu myeri ikumi namibiri mirabiriho?**

a) ee b) kwahi

20. **Wakaba wosiriho dozi zindi, kyani kyakuleterize kwosa mirundi gunyakusembayo?**

a) mirimo mi nkora b) nyebereiri c) nyalingi museiri hoi d) mibazii myalingi myahiho kwirwaru e) myandabirye kubibi f) bindi.........................

21. **Nani akuyambaho kumere mibazi?**

a) nyichala kwahi na yo b) mukori mwerawange c) mukali wange d) mwana wange

e) muzaire wange f) wondi......................................

22. **Watungiriho kintu kyensenya kiri kiibibi habwa buseiri bwa kasinsa kamunwereru?**

a) ee b) kwahi

23. **Nani giwawereiriho habwomi bwamu na kasinsa ka munwereru?**

a) mukali wange b) bana bange c) bankora na bo e) muntu wensenya wamu zigati f) muntu wahi

24. **Okiira kulara mirundi minghai hanze wa maka gamu mu sabiti gimwei**

a) kadii murundi gumwei gwahi b) murundi gumwei mu sabiiti

c)hagati wa mirundi 2 na 3 mu sabiiti d) kukira mu mirundi minei mu sabiiti

25. **Olimukitebe kindi kyensei kikukuyamba kumera mibazii?**

a) ee b) kwahi

26. **Okyegiri businsa bunene bwa munwereru bukusobora kuba bwamutalabaine ku bwomi bwamu**

a) ee b) kwahi

27. **Mu biibi, kyani kikuragiriza ngesu zamu zakunya sigara**

a) tinka nwangaho b) nyagereryeho gumwei rundi mibiiri

c)nyanwengi baitu mweri mirabiriho ndekiriho d) nchakanwambe na hatati

28. **Bakusomeseryeho ha mugasu gwa kuba na businsa budoli bwamunwereru?**

a) ee-------------------- b) kwahi

29. **Halubungo lwamu lunyakusembayo, wabuliriye kukori wa byabwomi ha bunene bwa kasinsa bwolinabwo habwire bubwo?**

a) ee b) kwahi

30. **Olina kwelalikiira kwensei bantu kwenga nkakwoli museiri wa buseiri bwa munwereru?**
    a) ee-------------- b) kwahi
31. **Okusobora kugeryagerya bwomi bwamu buwalingi nabwo otakatandikiri mibazii na buwatandikiri?**
    a) busemeiri b)mpinduka gyahai
    c) bukuhenekha buheneki d) nkusobora kwahi kukubwera
32. **Wosiriho kugyenda kwirwaru ha kiiro kibakuheiri mumyeri mukaaga mirabiriho?**
    a) ee b) kwahi
33. **Nsonga kyani zinyakuletererya kwosa kwamu**
    a)Nyakwatireni na miriimo b)bizibu bya ndibata c)nyebereiri
    d) ngesu zahai mubajanjabi e) bindi.......................................
34. **Wakoreseryeho mibazii myanzarwa kujanjaba buseiri bundi bwensei mumwaka gumwei gumalikiri**
    a) ee b) kwahi
35. **Olinamasuni gawatungiriho biibi mu myeri mukaaga mirabiriho? Bihika kumalika mubintu biwendyengi hoi na kunihira kukumalikamu**
    a) ee b) kwahi
36. **Mukugeryagerya kwamu, bujanajabi bwolinabwo hatati buli?**
    a) nabizibu binene hoi b) nabuzibu zibu budoli c) kizibu kyahai
37. Mirundi mingahi gyofunamy kyakunywa kirimu mwenge?
    a) Nkyalingi b) bulimweri undi kwahi c) hagati wa myeri mibiri kudwa mine d) mirundi mibiri kudwa misatu e) mirundi mine kudwa kugenda miso.
38. **Byakunywa bigahi byamuhendo byonywa mukiro kya muweek?**
    a) Kyakubanza oba kyakabiri b) kyakasatu oba kyakane c) kyakataano oba kyamukaaga d) musanju kudwa kyamwenda e) ikumi genda misio
39. **Mirundi mingahi nyonyamwo chupa mukaaga rundi kukiramu buli kiro?**
    a) Nya kwahi b) hansi wamweri gumwei c) buli mweri d) buli sabiiti e) buli kiro rundi bulikeire
40. **Mumwaka gumwe, mirundi mingahi niwambuka kugenda congo**
    a) Nyambuka kwahi b) hagati wamurundu gumwe namibiri c) kira mumurundi misatu.
41. **Mirundi mingahi nyolibata kuruga kumutanda gumwe kugenda kugundi?**
    a) Nyambuka kwahi b) hagati wamurundu gumwe namibiri c) kira mumurundi misatu.
42. **Bwoba olibeti, omarakayireki hanze nohokolera?**
    a) Hansi wamweri gumwe b) hagati wamweri gumwe na myeri mubiri c) kukira mu myeri misatu
43. **Okulengeseryamu tete kutalibanyizibwa kwa bijuma bya kasiisa kamunyereru namulimu gwokola?**
    a) Ntalibanyizibwa kwahi. b) Ntalibanyizibwa ho c) Ntalibanyizibwa hoi
44. **Oli mwikali wa nyamasaza ya Bulisa?**
    a) Eee b) kwahi
45. **Oli wahihangaki?**
    a) Munayuganda b) Munazairi c) zindi _________________________
46. **Wosirihona kumera mibazi habwambeera za murimu gwamu?**

a) Eee b) kwahi

KICHWEKA KYAKASATU: NSONGA ZAIRWARU ZIKWATEGEINI NA BUTAKENDERA BWA KASINSA KA BUSEIRI BWA MUNWERERO MU BADULU BAHANDU (MYAKA IKUMI NA MITANU NA KUGYENDA KWAKYENDI)

47. **Walemerweho kwiza hairwaru habwakuba irwaru lyalingi mu mbera zibiibi (mu bya buyoonjo)**
    a) ee b) kwahi
48. **Okupimapima teete irwaru kuba nirikuhegena bwire bukumala kurungi kusoboora nsonga zamu**
    a) burungi hoi b) manenineni c) bubiibi hoi
49. **Bakwebuliryeho ha kugonza kwamu ha mibazii mindi**
    a) ee b) kwahi
50. **Kyani kyokubazaho ha mbera zamu buwalingi nokyakatandiika kumera mibazii**

    a) bampambirize kutandiika b) bampeiri keire kwchweramu c) bind.......................

51. **Okusobora kukebera teete ntekaniza gairwaru mu nsita gyamu ha kasinsa ka munwereru**
    a) nsita gyahai b) nsita gili gidolidol c) nsita giliho ginene
52. **Bwere bwotwala kwirwaru okusobora kubusoobora teete**

    a) a) bunene hoi b) bunene c) budoli d) budoli hoi

53. **Byakwegesya kwa byabwomezi hairwaru lili**
    a) kwichala kwahamwei hoi b) kwahamwei kandi bakwirukya hoi c) kwikchala kwahiho
54. **Ha mutindo gwa byakubudabuda hairwaru lili**

    a) bwire bunene bugabwa kwetegereza kizibu kyenyini b) beirukya hoi

55. **48.Nziramu gya bajanajabi bakwiramyona bikaguzo byamu ha kasinsa ka munwereru**

    b) beiramwona kurungi hoi b) beiramwona kurungi c) beiramwona kwahi kurungi

56. **Bakuwereiriho kwemuka habwakuba mibazii myahai**

    a) ee b) kwahi

57. **Ha biiro bibakuhagana na milimo myamu**
    a) bidoli hoi kusigikira mulimo gwange b) bili birungi c) biba binene hoi kumpagana keire ka milimu myange
58. **Wezegwa teete ha bajanjabi ba kukolaho bulikiro**
    a) bensei bali na magezi b) bandi bali kwahi na magezi c) bensi begiri kwahi kasinsa ka munwereru kurungi
59. **Milingo myakujanjaba kasinsa kamunwereru bamisegerye hehi na kwamwamu rundi hokolera**

    a) ee b) kwahi

60. **Dactari wamu asoboiri kuba nakyetegeresya birugirimu kukebera kasinsa kamunwereru**

    a) ee b) kwahi

61. **Bwire bwa kutungiramwo birugiri mukukebera kasinsa ka munwereru bamali kukusika musai**
a) hansi wa mweri gumwei b) hagati wa mweri 1 na 2 c) kukira mu myeri mibiiri

62. **Hanyerabya gya bajanjabi**

a) bambokolera hoi b) bwire bundi bambokolera mbe c) bambokolera kwahi

63. **kukubulya ha bujuma busigeiriho mu mwaka gumwei gurabiriho**
a) bulikeire b) bwire bundi c) kabulya kwahi

64. **kubudabuda kunyakusembayo**

a) bakumperi nyenkenya b) bakutuheiri mu c) byense
