# Supplementary material for: Virological non-suppression among adult males attending HIV care services in the fishing communities in Bulisa district, Uganda
Source: PLoS One. 2023 Oct 19;18(10):e0293057. doi: 10.1371/journal.pone.0293057 (PMC10586650; doi:10.1371/journal.pone.0293057)
Supplement: S9 File — (PDF) [file pone.0293057.s009.pdf]

DATA ABSTRACTION TOOL

UNIQUE IDENTIFIER [ ]

This abstraction tool is to be applied to only adult males (15 years) residing in the fishing communities of Bulisa district with a viral load done 12 months prior to the date of data collection. The patient must be seeking care at one of the health facilities in Bulisa district

Data was extracted from the HIV clients blue chart. This is compiled in addition to the questionnaire above.

1. What is the patient's most current viral load? a) suppressed b) non suppressed
2. What is the patient's immediate previous viral load? Consider the immediate previous viral load
   a) Suppressed b) Non suppressed
3. What is the Patient's latest CD4 status? ____
4. Patient had TB diagnosed in the last one year? ____
5. What is the patient's current regimen? ____
6. Is the patient on first line or second line? ____
7. What is the patient's duration on ART? (insert enrolment date) ____________________________
8. What is the frequency of ART? ____
9. Baseline weight? _____________ Baseline Height _______________ BMI ____________________
10. Has patient changed regimen from the baseline regime? ___________________________________
11. What is the patient's current MUAC category (green/ yellow/red)? _________________________
12. What was the patient's baseline CD4? ____
13. What is the patient's latest adherence level (good, fair or poor)? ___________________________
14. What is the current WHO stage? ____
15. No of times has the patient interrupted treatment for more than 7 days in the last year? _____
16. Are there documented side effects in the last one year? ____________________________________
17. Baseline ART regimen? ____
18. Frequency of baseline regimen ____
