# Supplementary material for: Virological non-suppression among adult males attending HIV care services in the fishing communities in Bulisa district, Uganda
Source: PLoS One. 2023 Oct 19;18(10):e0293057. doi: 10.1371/journal.pone.0293057 (PMC10586650; doi:10.1371/journal.pone.0293057)
Supplement: S10 File — (PDF) [file pone.0293057.s010.pdf]

HEALTH FACILITY ASSESSMENT QUESTIONNAIRE FOR HEALTH WORKERS

UNIQUE IDENTIFIER ____________________

This Questionnaire is to be applied to only ART clinic in charges from health facilities in Bulisa district offering HIV treatment services

1. Are there days that the ART clinic remains unattended to by professional health workers
   a) Yes b) No
   If yes, how often does the clinic remain un-attended to?
   a) Less than twice a month b) 2-5 times a month c) more than 5times a month
2. Is there a schedule detailing topics for health education for patients attending the ART clinic?
   a) Yes b) No
3. Has the facility employed peer leaders living in the fishing communities to act as a link between the facility and patients from fishing communities?
   a) Yes b) No
4. In case you have identified peer leaders, have you oriented them on how they can support their fellow patients to have good outcomes?
   a) Yes b) No
5. Do you have staff designated to provide adherence counselling to patients that need it?
   a) Yes b) No
6. Have the staff providing counselling received formal training in counselling?
   a) Yes b) No
7. What method is used to assess adherence amongst HIV positive patients at this facility?
   a) Daily physical pill counts b) patient self-assessment of pills left c) health worker judgement d) others specify ____
8. Has this facility received any form of training in the management of HIV/AIDs basing on the latest guidelines?
   a) Yes b) No
9. How often do you receive refresher sessions on the guidelines of HIV?
   a) At least once a month b) At least once a quarter c) At least once every 6 months d) never
10. Are their instances you have missed assessing patients on time for viral load (This also includes repeat viral load) for patients on clinic days in the last year?
    a) Yes b) No

If yes, what could be the possible cause?

   a) Knowledge gap b) stock of required tools c) others specify

11. Are there clear communication channels that patients can use to consult in case of any challenges related to their treatment?
    a) Yes b) No
12. Have you come up with mechanisms to extend treatment services to patients who leave far from the facility?
    a) Yes b) No.

If yes, what mechanisms have you come up?

   a) CDDPS b) CCLAD groups c) others specify _________________________

13. What is the average time patients take at your facility on an ART clinic day?
    a) Less than 30 minutes b) 30 minutes – 1 hour c) 1 – 2 hours d) more than 2 hours
14. How often do you assess feedback at this facility from HIV patients residing in the fishing communities?
    a) Never b) once a month c) once a quarter.

15. Do you have a quality improvement team that meets routinely to discuss and address challenges among HIV patients resident in the fishing communities of Bulisa?
    a) Yes b) No
