# Supplementary material for: Virological non-suppression among adult males attending HIV care services in the fishing communities in Bulisa district, Uganda
Source: PLoS One. 2023 Oct 19;18(10):e0293057. doi: 10.1371/journal.pone.0293057 (PMC10586650; doi:10.1371/journal.pone.0293057)
Supplement: S11 File — (PDF) [file pone.0293057.s011.pdf]

MAKERERE UNIVERSITY

P.O. Box 7072 Kampala Uganda
Website: www.musph.ac.ug

Tel: 256 414 532207/543872/543437
Fax: 256 414 531807

COLLEGE OF HEALTH SCIENCES
SCHOOL OF PUBLIC HEALTH
HIGHER DEGREES, RESEARCH AND ETHICS COMMITTEE

2nd March, 2020

To whom it may concern

Dear Sir/Madam,

RE: PERMISSION TO CONDUCT RESEARCH

I hereby introduce to you Mr. Senteza Ignatius, a Master Student from Makerere University School of Public Health.

The student is required to conduct a research as a requirement for the award of the Master'sdegree. The title of the research study is: **"Factors Associated with Virological Non-Suppression among HIV positive Adult Males (15 years or Older) in the fishing communities of Bulisa District"**

The student presented the research proposal to the Makerere University School of Public Health, Higher Degrees, Research and Ethics Committee and has been granted approval to go ahead with data collection.

HIGHER DEGREES, RESEARCH AND ETHICS COMMITTEE
APPROVED ON
02 MAR 2020
P. O. BOX 7072, KAMPALA
MAKERERE UNIVERSITY SCHOOL OF PUBLIC HEALTH

Your support in this regard will be highly appreciated.

Yours sincerely,

Dr. Suzanne Kiwanuka
Chairperson: Higher Degrees, Research and Ethics Committee

MAKERERE **UNIVERSITY**

P.O. Box 7072 Kampala Uganda
Website: www.musph.ac.ug

Tel: 256 414 532207/543872/543437
Fax: 256 414 531807

COLLEGE OF HEALTH SCIENCES
SCHOOL OF PUBLIC HEALTH
HIGHER DEGREES, RESEARCH AND ETHICS COMMITTEE

2nd March, 2020

To whom it may concern

No objection

Dear Sir/Madam,

RE: ***PERMISSION TO CONDUCT RESEARCH***

I hereby introduce to you Mr. Senteza Ignatius, a Master Student from Makerere University School of Public Health.

The student is required to conduct a research as a requirement for the award of the Master'sdegree. The title of the research study is: **"Factors Associated with Virological Non-Suppression among HIV positive Adult Males (15 years or Older) in the fishing communities of Bulisa District"**

The student presented the research proposal to the Makerere University School of Public Health, Higher Degrees, Research and Ethics Committee and has been granted approval to go ahead with data collection.

Your support in this regard will be highly appreciated.

HIGHER DEGREES, RESEARCH & ETHICS COMMITTEE
APPROVED ON
0 2 MAR 2020
P. O. BOX 7072, KAMPALA
MAKERERE UNIVERSITY SCHOOL OF PUBLIC HEALTH

Yours sincerely,

Dr. Suzanne Kiwanuka
Chairperson: Higher Degrees, Research and Ethics Committee

MAKERERE UNIVERSITY

P.O. Box 7072 Kampala Uganda
Website: www.musph.ac.ug

Tel: 256 414 532207/543872/543437
Fax: 256 414 531807

COLLEGE OF HEALTH SCIENCES
SCHOOL OF PUBLIC HEALTH
HIGHER DEGREES, RESEARCH AND ETHICS COMMITTEE

2nd March, 2020

HIGHER DEGREES, RESEARCH AND ETHICS COMMITTEE
APPROVED ON
02 MAR 2020
P. O. BOX 7072, KAMPALA
MAKERERE UNIVERSITY SCHOOL OF PUBLIC HEALTH

Mr. Senteza Ignatius

Master student, (2016/HD07/2375U

School of Public Health, Makerere University

Re: Approval of a research Proposal titled: Factors Associated with Virological Non-suppression among HIV Positive Adult Males (15 Years or Older) in the Fishing Communities of Bulisa District

This is to inform you that the Higher Degrees, Research and Ethics Committee (HDREC) has approved your study documents for the above referenced research study.

Note that your study was first approved by the HDREC on **3rd/03/2020**, and therefore approval expires at every annual anniversary of this approval date. The current approval is therefore valid until: **2nd/02/2021**

Continued approval is conditional upon your compliance with the following requirements:

1) No other consent form(s), questionnaire and/or advertisement documents should be used. The consent form(s) must be signed by each subject prior to initiation of any protocol procedures. In addition, each subject must be given a copy of the signed consent form.

2) All protocol amendments and changes to other approved documents must be submitted to HDREC and not be implemented until approved by HDREC except where necessary to eliminate apparent immediate hazards to the study subjects.

3) Significant changes to the study site and significant deviations from the research protocol and all unanticipated problems that may involve risks or affect the safety or welfare of subjects or others, or that may affect the integrity of the research must be promptly reported to HDREC.

4) For Masters Students in the School of Public Health, you are required to submit 2 copies of your proposal plus a letter of intention to submit a dissertation giving a period of 3 months to the School of Graduate Studies before you commence data collection

- Please complete and submit reports to HDREC as follows:

a) Renewal of the study approval – complete and return the continuing Review Report – Renewal Request (Form 404A) at least 60 days prior to the expiration of the approval period. The study cannot continue until re-approved by HDREC.

b) Completion, termination, or if not renewing the project – send a final report within 90 days upon completion of the study.

Yours sincerely,

HIGHER DEGREES, RESEARCH AND ETHICS COMMITTEE
APPROVED ON
0 2 MAR 2020
P. O. BOX 7072, KAMPALA
MAKERERE UNIVERSITY SCHOOL OF PUBLIC HEALTH

Dr. Suzanne Kiwanuka

Chairperson: Higher Degrees, Research and Ethics Committee
