# Supplementary material for: Virological non-suppression among adult males attending HIV care services in the fishing communities in Bulisa district, Uganda
Source: PLoS One. 2023 Oct 19;18(10):e0293057. doi: 10.1371/journal.pone.0293057 (PMC10586650; doi:10.1371/journal.pone.0293057)
Supplement: S13 File — (DOCX) [file pone.0293057.s013.docx]

| **CODE** |  |
| --- | --- |
| **INDIVIDUAL FACTORS** | |
|  | **Current viral load** |
| 0 | Suppressed |
| 1 | Non supressed |
|  | **Immediate previous viral load** |
| 0 | Suppressed |
| 1 | Non suppressed |
|  | **Latest CD4** |
| 0 | Less than 200 |
| 1 | 200-500 |
| 2 | Greater than 500 |
|  | **Diagnosed with TB in the last one year** |
| 0 | No  yes |
| 1 | Yes |
|  | **Current ART regimen** |
| 0 | TDF/3TC/EFV |
| 1 | TDF/3TC/DTG |
| 2 | TDF/3TC/ATV/r |
| 3 | AZT/3TC/NVP |
| 4 | Other second line |
| 5 | Other first line |
|  | **Line of current regimen** |
| 0 | First |
| 1 | Second/ third |
|  | **Duration on ART** |
| 0 | Less than 24 months |
| 1 | 24-50 months |
| 2 | Greater than 50 months |
|  | **Frequency of current ART regimen** |
| 0 | Once daily |
| 1 | Twice daily |
|  | **Ever changed regimen from baseline** |
| 0 | yes |
| 1 | No |
|  |  |
|  | **Body mass index** |
| 0 | Less than 18.5 |
| 1 | 18.5 to 24.9 |
| 2 | Greater than or equal to 25.0 |
|  | **Mid upper arm circumference** |
| 0 | Green |
| 1 | Yellow/ red |
|  | **Baseline CD4** |
| 0 | Less than 200 |
| 1 | 200-500 |
| 2 | Greater than 500 |
|  | **Documented adherence level** |
| 0 | Good |
| 1 | Fair |
| 2 | Poor |
|  | **Clinical Stage** |
| 0 | I |
| 1 | II |
| 2 | III and IV |
|  | **More than 7 days appointment interruption in last 12 months** |
| 0 | 0 |
| 1 | 1 to 3 |
| 2 | More than 3 |
|  | **Side effects** |
| 0 | No |
| 1 | yes |
|  | **Baseline ART** |
| 0 | AZT/3TC/NVP |
| 1 | AZT/3TC/EFV |
| 2 | TDF/3TC/EFV |
| 3 | TDF/3TC/DTG |
| 4 | Other regimens |
|  | **Frequency of baseline regimen** |
| 0 | Once daily |
| 1 | Twice daily |
|  | **Age category** |
| 0 | Greater than 50 years |
| 1 | 26 to 50 |
| 2 | 15 to 25 |
|  |  |
|  | **Marital status** |
| 0 | Single |
| 1 | Divorced |
| 2 | Married |
| 3 | Cohabiting |
| 4 | Widowed |
|  | **Ability to read and write** |
| 0 | No |
| 1 | Yes |
|  | **Highest level of education** |
| 0 | No schooling |
| 1 | Completed primary |
| 2 | Completed secondary |
| 3 | Some Primary |
| 4 | Some secondary |
|  | **Occupation** |
| 0 | Others jobs |
| 1 | Fishing / trading in fish |
| 2 | Not employed |
|  | **Average income** |
|  |  |
| 0 | > UGX 20,000 |
| 1 | < UGX 10,000 |
| 2 | UGX 10,000 – 20,000 |
|  | **Religion** |
| 0 | Catholic |
| 1 | Born again |
| 2 | Muslim |
| 3 | Others |
| 4 | Protestant |
|  | **Time to facility** |
| 0 | Less than 1 hour |
| 1 | 1-2 hours |
| 2 | 2 hours |
|  | **Transport costs** |
| 0 | 2000-5000 |
| 1 | 5000-10,000 |
| 2 | Greater than 10,0000 |
|  | **Missed appointment because of transport** |
| 0 | No |
| 1 | Yes |
|  | **Meals per day** |
| 0 | 1 meal |
| 1 | 2-3 meals |
| 2 | More than 3 meals |
|  | **Missed ART because of food** |
| 0 | No |
| 1 | Yes |
|  | **No of sexual partners** |
| 0 | None |
| 1 | Only one |
| 2 | More than one |
|  | **Condom use** |
| 0 | Never |
| 1 | Once in a while |
| 2 | Every time |
|  | **Partner HIV status** |
| 0 | Don’t know |
| 1 | Negative |
| 2 | Positive |
|  | **Partner on ART** |
| 0 | No |
| 1 | Yes |
|  | **Partner getting ART from same facility** |
| 0 | No |
| 1 | Yes |
|  | **Knowledge of partner suppression** |
| 0 | Suppressed |
| 1 | Non suppressed |
| 2 | Don’t know |
|  | **Missed taking ART in last 12 months** |
| 0 | No |
| 1 | yes |
|  | **Reason for missing ART in last 12 months** |
| 0 | Transport / travel |
| 1 | Forgot |
| 2 | Was too sick |
| 3 | Lack of food |
| 4 | The work I do |
| 5 | Other reasons |
|  | **Treatment supporter** |
| 0 | Don’t have |
| 1 | Work mate |
| 2 | Wife |
| 3 | Any other family member |
|  | **Suffered a stressful life event following HIV diagnosis** |
| 0 | No |
| 1 | Yes |
|  | **HIV disclosure** |
| 0 | No one |
| 1 | Any other family member |
| 2 | Work mate/ friend/neighbour |
| 3 | Wife |
|  | **Frequency of spending nights outside home** |
| 0 | Never |
| 1 | Once a week |
| 2 | 2-3 times a week |
| 3 | More than 3 times a week |
|  | **Awareness of dangers of viral load** |
| 0 | No |
| 1 | Yes |
|  | **Smoking behaviour** |
| 0 | Never |
| 1 | Used to some but quit |
| 2 | Tried once and stopped |
| 3 | Still smoke up to now |
|  | **Inquisitive about vial load status** |
| 0 | No |
| 1 | Yes |
|  | **Afraid about disclosing HIV status** |
| 0 | No |
| 1 | Yes |
|  | **Health status after starting ART** |
| 0 | About the same |
| 1 | Can’t tell |
| 2 | Getting worse |
| 3 | Improved |
|  | **Missed clinic appointment in last 6 months** |
| 0 | No |
| 1 | Yes |
|  | **Reason for missing appointment** |
| 0 | Forgot |
| 1 | Engaged in work |
| 2 | Feeling sick/ side effects |
| 3 | Travelled/ transport |
|  | **Use of traditional medicines** |
| 0 | No |
| 1 | Yes |
|  | **Lost interest in pleasurable activities** |
| 0 | No |
| 1 | Yes |
|  | **Safety perception of HIV related drugs** |
| 0 | Have no problem |
| 1 | Have some problems |
| 2 | Have many problems |
|  | **Hazardous use of alcohol** |
| 0 | Non-hazardous use |
| 1 | Hazardous use |
|  | **Frequency of cross over to Congo in a year** |
| 0 | Never |
| 1 | Once or twice |
| 2 | More than twice |
|  | **Frequency of moving between landing sites in a year** |
| 0 | More than twice |
| 1 | Once or twice |
| 2 | Never |
|  | **Duration spend outside usual workplace due to mobility** |
| 0 | Less than 1 month |
| 1 | 1 to 2 months |
| 2 | Three or more months |
|  | **ARVs and stability at work** |
| 0 | No disruption |
| 1 | Disruption |
|  | **Permanent resident of Bulisa** |
| 0 | No |
| 1 | Yes |
|  | **Nationality** |
| 0 | Non Ugandan |
| 1 | Ugandan |
|  |  |
|  | **Missed ARVs because of work** |
| 0 | No |
| 1 | Yes |
| HEALTH FACILITY FACTORS | |
|  | **Participant’s facility for treatment** |
| 0 | Facility at HCII level |
| 1 | Facility at HC III level |
| 2 | Facility at HC IV level |
| 3 | Facility at Hospital level |
|  |  |
|  |  |
|  | **Taught about importance of viral load suppression** |
| 0 | No |
| 1 | Yes |
|  | **Belonging to a treatment support group** |
| 0 | Yes |
| 1 | No |
|  | **Ever missed appointment due to facility set up/ hygiene** |
| 0 | No |
| 1 | Yes |
|  | **Assessment of attention given to concern expression** |
| 0 | Very good |
| 1 | Fair |
| 2 | Good |
|  | **Engaged in selecting treatment options** |
| 0 | No |
| 1 | Yes |
|  | **Experience when starting ART** |
| 0 | forced |
| 1 | Given time to make a decision |
|  | **Assessment of confidentiality at facility** |
| 0 | No confidentiality |
| 1 | Some confidentiality |
| 2 | Maximum confidentiality |
|  | **Waiting time at facility** |
| 0 | Very short |
| 1 | Short |
| 2 | long |
| 3 | Very long |
|  | **Quality of health education talks** |
| 0 | Do not happen at all |
| 1 | Less interactive and rushed |
| 2 | Interactive |
|  | **Quality of counselling sessions** |
| 0 | Given enough time |
| 1 | rushed |
|  | **Satisfaction of answering HIV related questions** |
| 0 | Very satisfied |
| 1 | Satisfied |
| 2 | Not very satisfied |
|  | **Ever missed drugs because of drug stock outs** |
| 0 | No |
| 1 | Yes |
|  | **Quality of clinical appointments and work** |
| 0 | Long enough |
| 1 | They are just fine |
| 2 | Too short |
|  | **Perceived competency of health workers** |
| 0 | All are knowledgeable |
| 1 | Some lack knowledge |
| 2 | All lack knowledge |
|  | **HIV services extended near work** |
| 0 | Yes |
| 1 | No |
|  | **Made to understand my viral load** |
| 0 | No |
| 1 | Yes |
|  | **Viral load turnaround time** |
| 0 | Can’t tell |
| 1 | 1 to 2 months |
| 2 | More than 2 months |
| 3 | Less than 1 month |
|  | **Health work gentleness** |
| 0 | Never shout |
| 1 | Sometimes shout at me |
| 2 | Always shout at me |
|  | **Pill balance assessment** |
| 0 | Every time |
| 1 | Some times |
| 2 | Never been asked |
|  | **Counselling session** |
| 0 | Given individually |
| 1 | Given in a group |
| 2 | Both individual and group |
| 3 | Never been given |
