# Supplementary material for: Virological non-suppression among adult males attending HIV care services in the fishing communities in Bulisa district, Uganda
Source: PLoS One. 2023 Oct 19;18(10):e0293057. doi: 10.1371/journal.pone.0293057 (PMC10586650; doi:10.1371/journal.pone.0293057)
Supplement: Attachment — Submitted filename: Review of virological non-suppression among adult males attending HIV cares services in Uganda.docx [file pone.0293057.s014.docx]

**Review of “Virological non-suppression among adult males attending HIV care services in the fishing communities in Bulisa district, Uganda”**

Summary of review

Thank you for the opportunity to review this paper that outlined HIV virologic non-suppression and covariates. This is an important piece of work that adds to understanding one of the more vulnerable groups in Uganda. Overall, the manuscript was well-written. I have some specific points of clarification mainly in the Methods Section. Additionally, the Discussion section could be further expanded to better put the current findings into the broader research context.

Additional questions and suggestions are offered by manuscript section:

Overall

1. Please use people first language, for example instead of writing HIV positive, please change to people with HIV or men with HIV.

Introduction

1. It would be helpful for readers if you separated the one big Introduction paragraph into several smaller paragraphs.
2. The authors state that the HIV prevalence in the Bulisa district is 5.1%, but this is lower than the 2020 country-wide prevalence of 5.4% according to UNAIDS, please double-check the data and sources. It would be helpful to include years and sources in the text when feasible given that in this one sentence there appears to be comparisons among three different sources. It would also be helpful for the reader if you compared the Bulisa district HIV prevalence to the overall country-wide HIV prevalence for context.

“In a study carried out in Uganda using national programme data in 2017, the prevalence of nonsuppression among males was 13% [15]. However, in Bulisa district, with 14.2% of the population engaged in fishing business [16] and HIV prevalence of 5.1% [17], the

prevalence of virological non-suppression amongst all adult males for the same period was much higher at 20%.”

Methods

1. Under “study setting”, when describing the different management pathways for different patients, I would use the formal terminology – such as, “differentiated care models”.
2. Under “study setting”, please define facility based improved management.
3. Under “study design”, the first sentence is likely missing a word, “We conducted a cross-sectional *** utilising patients records and interviews to determine the prevalence of virological non-suppression and factors associated with virological nonsuppression among adult males, resident in the fishing communities of Bulisa district.”
4. Under “study design” please clarify whether interviews were also done with MWH participants.
5. Under “participant selection”, the following sentence is likely missing a word, “One health worker per facility was purposively selected for the face to *** interviews.”
6. Under “data collection and management”, please dscribe in more detail how adherence was categorized – was this self-report, a questionnaire, etc.?
7. Under “data analysis”, what was the definition of virologic non-suppression? What was the virologic threshold? What was the definition of “up to date” viral load – what was the viral load timing cut-off point for study inclusion?
8. Under “ethical considerations”, please also include if Uganda National Council of Science and Technology approval was obtained.
9. Under “data analysis”, please explain the rationale for when the authors chose to use means vs. medians. I would also outline that proportions and frequencies were described.
10. It would be helpful for the reader for a more detailed explanation of what the authors mean by landing sites – are these sites still in the same fishing villages – are they farther away? What are the implications of moving between different landing sites?
11. Please describe in greater details the questionnaire procedures with study staff. How were staff chosen to interview? How was the questionnaire designed?

Results

1. Please clarify over what amount of time participants reported number of sexual partners.
2. Under “Factors associated with virological non-suppression”, it would be more clear when comparing VL suppression in relation to income, if you compared the lower wage participants as baseline to the higher wage participants (so that your PR is >1 – similar to the other variables you have discussed). I would recommend this for any PR that is reported as <1 in the results.

Discussion

1. Paragraph 2, the authors state that men earning more money were less likely to have virological suppression. This appears to be the opposite of the data presented.
2. Consider “social desirability bias” to explain this concept – “Information bias could have also resulted from desirability by some participants responding to some questions like those assessing sexual behavior with reservations.”
3. I would recommend developing the first five or so paragraphs discussing the pertinent covariates of non-virologic suppression. For each point that you bring up, please expand the comparison to the literature and the implications for your specific research site.
4. An additional limitation could be the lack of back translation from the local language to English if this was not done.

Figures/Tables

1. Figure 1 has typos, please edit – ex: “Dint meet eligibility criteria 367”
