# Supplementary material for: Virological non-suppression among adult males attending HIV care services in the fishing communities in Bulisa district, Uganda
Source: PLoS One. 2023 Oct 19;18(10):e0293057. doi: 10.1371/journal.pone.0293057 (PMC10586650; doi:10.1371/journal.pone.0293057)
Supplement: Attachment — Submitted filename: Response to Reviewers.docx [file pone.0293057.s015.docx]

| Journal Requirements: | REMARKS | |
| --- | --- | --- |
| When submitting your revision, we need you to address these additional requirements. | Noted | |
| 1. Please ensure that your manuscript meets PLOS ONE's style requirements, including those for file naming. The PLOS ONE style templates can be found at  <https://journals.plos.org/plosone/s/file?id=wjVg/PLOSOne_formatting_sample_main_body.pdf> and  <https://journals.plos.org/plosone/s/file?id=ba62/PLOSOne_formatting_sample_title_authors_affiliations.pdf> |  | |
| 2. You indicated that you had ethical approval for your study. In your Methods section, please ensure you have also stated whether you obtained consent from parents or guardians of the minors included in the study or whether the research ethics committee or IRB specifically waived the need for their consent. | Ascent from guardians of participants aged 15 to 18 years was obtained. This has been included under the methodology section. | |
| 3. We note that the grant information you provided in the ‘Funding Information’ and ‘Financial Disclosure’ sections do not match. |  | |
| When you resubmit, please ensure that you provide the correct grant numbers for the awards you received for your study in the ‘Funding Information’ section. | This has been rectified | |
| 4. Thank you for stating in your Funding Statement: "SI was partly funded by the Gilead foundation BC was partly funded by the Fogarty International Centre, National Institute of Health (grant# 2D43TW009771-06 “HIV and co-infections in Uganda") The management of people living with HIV was partly supported by the Presidents Emergency Plan for AIDS Relief through the United States Centers for Disease Control (CDC) and Prevention and the terms of cooperative agreement number NU2GGH001294-03-05" | Funding statement amended | |
| Please provide an amended statement that declares *all* the funding or sources of support (whether external or internal to your organization) received during this study, as detailed online in our guide for authors at <http://journals.plos.org/plosone/s/submit-now>.  Please also include the statement “There was no additional external funding received for this study.” in your updated Funding Statement.  Please include your amended Funding Statement within your cover letter. We will change the online submission form on your behalf. |  | |
| 5. Thank you for stating the following financial disclosure: "SI was partly funded by the Gilead foundation BC was partly funded by the Fogarty International Centre, National Institute of Health (grant# 2D43TW009771-06 “HIV and co-infections in Uganda") The management of people living with HIV was partly supported by the Presidents Emergency Plan for AIDS Relief through the United States Centers for Disease Control (CDC) and Prevention and the terms of cooperative agreement number NU2GGH001294-03-05" | This has been rectified in the cover letter. | |
| Please state what role the funders took in the study.  If the funders had no role, please state: "The funders had no role in study design, data collection and analysis, decision to publish, or preparation of the manuscript. If this statement is not correct you must amend it as needed.  Please include this amended Role of Funder statement in your cover letter; we will change the online submission form on your behalf. | This has been included in the cover letter. | |
| 6. Please include captions for your Supporting Information files at the end of your manuscript, and update any in-text citations to match accordingly. Please see our Supporting Information guidelines for more information: <http://journals.plos.org/plosone/s/supporting-information>. | These have been included. | |
| [Note: HTML markup is below. Please do not edit.]  Reviewers' comments:  Reviewer's Responses to Questions |  | |
| **Comments to the Author**  1. Is the manuscript technically sound, and do the data support the conclusions?  The manuscript must describe a technically sound piece of scientific research with data that supports the conclusions. Experiments must have been conducted rigorously, with appropriate controls, replication, and sample sizes. The conclusions must be drawn appropriately based on the data presented. |  | |
| Reviewer #1: Partly |  | |
| Reviewer #2: Yes |  | |
|  |  | |
| 2. Has the statistical analysis been performed appropriately and rigorously? |  | |
| Reviewer #1: No |  | |
| Reviewer #2: I Don't Know |  | |
|  |  | |
| 3. Have the authors made all data underlying the findings in their manuscript fully available?  The [PLOS Data policy](http://www.plosone.org/static/policies.action#sharing) requires authors to make all data underlying the findings described in their manuscript fully available without restriction, with rare exception (please refer to the Data Availability Statement in the manuscript PDF file). The data should be provided as part of the manuscript or its supporting information, or deposited to a public repository. For example, in addition to summary statistics, the data points behind means, medians and variance measures should be available. If there are restrictions on publicly sharing data—e.g. participant privacy or use of data from a third party—those must be specified. |  | |
| Reviewer #1: Yes |  | |
| Reviewer #2: Yes |  | |
|  |  | |
| 4. Is the manuscript presented in an intelligible fashion and written in standard English?  PLOS ONE does not copyedit accepted manuscripts, so the language in submitted articles must be clear, correct, and unambiguous. Any typographical or grammatical errors should be corrected at revision, so please note any specific errors here. |  | |
| Reviewer #1: No |  | |
| Reviewer #2: Yes |  | |
|  |  | |
| 5. Review Comments to the Author  Please use the space provided to explain your answers to the questions above. You may also include additional comments for the author, including concerns about dual publication, research ethics, or publication ethics. (Please upload your review as an attachment if it exceeds 20,000 characters) |  | |
| Reviewer #1: Summary: This manuscript summarizes findings of an interesting analysis of predictors of viral non-suppression among men in fishing communities in Uganda, a high-risk population.  **Major comments:** | Comment well appreciated. | |
| 1. The outcome of interest of viral non-suppression should be defined. It is not clear what viral load threshold is being used in this outcome determination (i.e. not detected, 50 copies/mL, 1,000 copies/mL). | Addressed in the edited document. | |
| 2. The multivariable model includes 15 predictors, many of which are multi-level categorical variables. Given that only 84 participants had non-suppression, there may be a risk of having overfit the model with too many predictors. The authors should provide justification for this or reconsider the variables that are included in the final model. | Major edits were done following this comment. 88 participants had virological non-suppression. To avoid the risk of overfitting the model as guided, the 1 in 10 to rule was used and only 9 independent variables were used. (8.8 rounded off to 9). Several variables in the previous model were dropped and some were replaced in the final model.  References   1) Interactive Text Book on Clinical Symptom Research   [Chapter 8: Statistical Models for Prognostication: Problems with Regression Models (archive.org)](https://web.archive.org/web/20041031140843/http:/painconsortium.nih.gov/symptomresearch/chapter_8/sec8/cess8pg2.htm).   2) Steyerberg EW, Eijkemans MJ, Harrell Jr FE, Habbema JD. Prognostic modelling with logistic regression analysis: a comparison of selection and estimation methods in small data sets. Statistics in medicine. 2000 Apr 30;19(8):1059-79. 3) Steyerberg EW, Eijkemans MJ, Harrell Jr FE, Habbema JD. Prognostic modeling with logistic regression analysis: in search of a sensible strategy in small data sets. Medical Decision Making. 2001 Feb;21(1):45-56. | |
| 3. Would consider reframing conclusions to be more generalizable to a regional or global audience, rather than specific implications for the district health office for the area in which the study took place. | Conclusions and recommendations have been reframed as guided. | |
| Minor comments: 1. The authors should use person-centered language in describing HIV status. Specifically, rather than referring to HIV positive adults, the authors should use the term “people with HIV.” | This was addressed throughout the whole document. | |
| 2. Methods – Study design: Further detail on the participant and healthworker interview regarding the health facilities would be helpful. Specifically, were interviews standardized by questionnaire, or were they open-ended and then coded using qualitative methodology? These methods and the factors explored should be more clearly explained. | Exploration on the factors considered was done.  Elaboration on the interviewing process included. | |
| 3. Methods – Data collection: Not all factors explored in the regression analyses are explained in the methods section. Authors should note if adherence data are based on self-report, clinician assessment, or pill count. It would be also important to understand at what time point the predictors of interest of clinic and ART regimen are related to. For example, if ART regimen is listed as second-line, is that because a second-line regimen was prescribed at the visit when an elevated viral load was acted on, or was the viral load collected while on a second-line regimen? The same would be true for clinic, given that patients with virologic failure may be referred to higher level health centers. | All factors explored in the regression analysis have been explained in the methodology section A viral load within a period of 12 months from the time of sample collection was compiled for this study. The relationship of this viral load with the duration on second line regimen was compiled for this study. So, it is hard to tell whether a high viral load was related to deciding to switch to second line or it applied to a patient who had been on second line for atleast 6 month.  This is one of the indicators that were replaced while reducing the number of variables at multivariable analysis | |
| 4. Methods – Data analysis: It seems that a complete case analysis strategy was employed, rather than imputing missing data. If that is correct, this should be stated. | Case based analysis was used | |
| 5. Results – When referring to 829 men in HIV care in Bulisa district, is this fully inclusive of all patients at all health facilities in the district, or only a subset? It would also be helpful to understand why 367 were excluded (which inclusion criteria were not met?). | Inclusion criteria was included in the consort diagram. | |
| 6. Discussion – The authors note that longer duration between appointments was beneficial, but these data are not clearly located in the results table. Does this refer to the predictor labeled “clinical appointments and work”? The wording of the response options could be confused with referring to the length of the clinical visit itself, rather than the time interval between visits. | Clinical appointments were assessed as long or short according to the participants perception.  Time interval between visits is much better as suggested for consideration under discussion. | |
| 7. Discussion – The authors recommend the establishment of savings groups in fishing communities to improve treatment adherence. However, this isn’t discussed elsewhere in this analysis/manuscript. | Saving groups had been suggested as a way to boost the income of participants since average income had an association with virological non-suppression. However, the guidance on the wording is noted for consideration. | |
| 8. Figure 1 – It would be helpful to be consistent with inclusion of percentages and numbers of participants. | All percentages have been included in the table | |
| 9. Results – The authors should explain why age and regimen change were not included in the multivariable model. | Age has been included in the revised model as one of the universal confounders.  At Multivariable analysis, inclusion of variables was mainly determined by the uniqueness in relation to fishing communities. Variables with a p-value less than 0.25 were included and eliminated until a better model was obtained. Regimen change was one of them. Multicollinearity is also another reason why it was eliminated. | |
| 10. The manuscript should be carefully reviewed and edited to correct typos, sentence structure errors, and spelling errors throughout. |  | |
| Reviewer #2: Review of “Virological non-suppression among adult males attending HIV care services in the fishing communities in Bulisa district, Uganda”  Summary of review  Thank you for the opportunity to review this paper that outlined HIV virologic non-suppression and covariates. This is an important piece of work that adds to understanding one of the more vulnerable groups in Uganda. Overall, the manuscript was well-written. I have some specific points of clarification mainly in the Methods Section. Additionally, the Discussion section could be further expanded to better put the current findings into the broader research context.  Additional questions and suggestions are offered by manuscript section: |  | |
| Overall 1. Please use people first language, for example instead of writing HIV positive, please change to people with HIV or men with HIV. | This was addressed throughout the whole document. | |
| Introduction 1. It would be helpful for readers if you separated the one big Introduction paragraph into several smaller paragraphs. | This was done | |
| 2. The authors state that the HIV prevalence in the Bulisa district is 5.1%, but this is lower than the 2020 country-wide prevalence of 5.4% according to UNAIDS, please double-check the data and sources. It would be helpful to include years and sources in the text when feasible given that in this one sentence there appears to be comparisons among three different sources. It would also be helpful for the reader if you compared the Bulisa district HIV prevalence to the overall country-wide HIV prevalence for context. | This was rectified | |
| “In a study carried out in Uganda using national programme data in 2017, the prevalence of non-suppression among males was 13% [15]. However, in Bulisa district, with 14.2% of the population engaged in fishing business [16] and HIV prevalence of 5.1% [17], the prevalence of virological non-suppression amongst all adult males for the same period was much higher at 20%.” | This was rectified | |
| Methods 1. Under “study setting”, when describing the different management pathways for different patients, I would use the formal terminology – such as, “differentiated care models”. | Adopted as guided | |
| 2. Under “study setting”, please define facility based improved management.  3. Under “study design”, the first sentence is likely missing a word, “We conducted a cross-sectional *** utilising patients records and interviews to determine the prevalence of virological non-suppression and factors associated with virological nonsuppression among adult males, resident in the fishing communities of Bulisa district.” | This was rectified | |
| 4. Under “study design” please clarify whether interviews were also done with MWH participants. | MWH not clear to me. But interview section was rectified | |
| 5. Under “participant selection”, the following sentence is likely missing a word, “One health worker per facility was purposively selected for the face to *** interviews.” | This was rectified | |
| 6. Under “data collection and management”, please dscribe in more detail how adherence was categorized – was this self-report, a questionnaire, etc.? | Categorization of adherence was included | |
| 7. Under “data analysis”, what was the definition of virologic non-suppression? What was the virologic threshold? What was the definition of “up to date” viral load – what was the viral load timing cut-off point for study inclusion? | All this has been clarified under data collection and management. | |
| 8. Under “ethical considerations”, please also include if Uganda National Council of Science and Technology approval was obtained. | This study was done as part of the fulfilments required for the awards of Master’s degree in Public health at Makerere University.  The university/ IRB has the responsibility to submit a list of student’s researches to UNCST. My letter for approval is attached here.  | |
| 9. Under “data analysis”, please explain the rationale for when the authors chose to use means vs. medians. I would also outline that proportions and frequencies were described. |  |  |
|  | Mean (SD) was used as a measure of central tendency for age since this had a normal distribution. Duration on ART had a skewed distribution hence median (IQR) was used as the measure of central tendency | |
| 10. It would be helpful for the reader for a more detailed explanation of what the authors mean by landing sites – are these sites still in the same fishing villages – are they farther away? What are the implications of moving between different landing sites? | Landing sites have been defined under study setting.  The phrase “landing sites” has been edited to “fish landing sites” | |
| 11. Please describe in greater details the questionnaire procedures with study staff. How were staff chosen to interview? How was the questionnaire designed? | This has been addressed under study design and participants selection. | |
| Results 1. Please clarify over what amount of time participants reported number of sexual partners. | This has been addressed | |
| 2. Under “Factors associated with virological non-suppression”, it would be more clear when comparing VL suppression in relation to income, if you compared the lower wage participants as baseline to the higher wage participants (so that your PR is >1 – similar to the other variables you have discussed). I would recommend this for any PR that is reported as <1 in the results. | This was considered for all other variables that has PR < 1 included at multivariable analysis. | |
| Discussion 1. Paragraph 2, the authors state that men earning more money were less likely to have virological suppression. This appears to be the opposite of the data presented. | Average income is one of the variables that were dropped when cutting down the number of variables from 15 to 9 at multivariable analysis. Justification for cutting down the variables was considered to avoid the risk of over fitting the model following the guidance from reviewer one but also from the reviewed literature. | |
| 2. Consider “social desirability bias” to explain this concept – “Information bias could have also resulted from desirability by some participants responding to some questions like those assessing sexual behavior with reservations.” | Indeed, social desirability explains the responses to sexual behavior questions better as guided. This has been considered in the manuscript.  [Social Desirability Bias \| SpringerLink](https://link.springer.com/referenceworkentry/10.1007/978-94-007-0753-5_2746) | |
| 3. I would recommend developing the first five or so paragraphs discussing the pertinent covariates of non-virologic suppression. For each point that you bring up, please expand the comparison to the literature and the implications for your specific research site. | This was rectified under discussion | |
| 4. An additional limitation could be the lack of back translation from the local language to English if this was not done. | This has been considered. | |
| Figures/Tables 1. Figure 1 has typos, please edit – ex: “Dint meet eligibility criteria 367” | Typos have been revised in the entire document | |
| 6. PLOS authors have the option to publish the peer review history of their article ([what does this mean?](https://journals.plos.org/plosone/s/editorial-and-peer-review-process#loc-peer-review-history)). If published, this will include your full peer review and any attached files.   If you choose “no”, your identity will remain anonymous but your review may still be made public. | No objection | |
| **Do you want your identity to be public for this peer review?** For information about this choice, including consent withdrawal, please see our [Privacy Policy](https://www.plos.org/privacy-policy). |  | |
| Reviewer #1: No |  | |
| Reviewer #2: No |  | |
|  |  | |
|  |  | |
| [NOTE: If reviewer comments were submitted as an attachment file, they will be attached to this email and accessible via the submission site. Please log into your account, locate the manuscript record, and check for the action link "View Attachments". If this link does not appear, there are no attachment files.]  While revising your submission, please upload your figure files to the Preflight Analysis and Conversion Engine (PACE) digital diagnostic tool, <https://pacev2.apexcovantage.com/>. PACE helps ensure that figures meet PLOS requirements. To use PACE, you must first register as a user. Registration is free. Then, login and navigate to the UPLOAD tab, where you will find detailed instructions on how to use the tool. If you encounter any issues or have any questions when using PACE, please email PLOS at [figures@plos.org](mailto:figures@plos.org). Please note that Supporting Information files do not need this step. |  | |
