# Supplementary material for: Virological non-suppression among adult males attending HIV care services in the fishing communities in Bulisa district, Uganda
Source: PLoS One. 2023 Oct 19;18(10):e0293057. doi: 10.1371/journal.pone.0293057 (PMC10586650; doi:10.1371/journal.pone.0293057)
Supplement: Attachment — Submitted filename: Response to reviewers.docx [file pone.0293057.s016.docx]

**Comments to the Author addressed**

| **Comment** | **Response by the author.** |
| --- | --- |
| 1. If the authors have adequately addressed your comments raised in a previous round of review and you feel that this manuscript is now acceptable for publication, you may indicate that here to bypass the “Comments to the Author” section, enter your conflict of interest statement in the “Confidential to Editor” section, and submit your "Accept" recommendation.   Reviewer #2: All comments have been addressed | We are glad that this was satisfactorily addressed in our first set of revisions |
| Is the manuscript technically sound, and do the data support the conclusions?  The manuscript must describe a technically sound piece of scientific research with data that supports the conclusions. Experiments must have been conducted rigorously, with appropriate controls, replication, and sample sizes. The conclusions must be drawn appropriately based on the data presented  Reviewer #2: Yes | We are glad that this was satisfactorily addressed in our first set of revisions |
| 3. Has the statistical analysis been performed appropriately and rigorously?  Reviewer #2: I Don't Know | To the best our knowledge, we put out the best output as guided by reviewer one in the first set of revisions. |
| 4. Have the authors made all data underlying the findings in their manuscript fully available?  The [PLOS Data policy](http://www.plosone.org/static/policies.action#sharing) requires authors to make all data underlying the findings described in their manuscript fully available without restriction, with rare exception (please refer to the Data Availability Statement in the manuscript PDF file). The data should be provided as part of the manuscript or its supporting information, or deposited to a public repository. For example, in addition to summary statistics, the data points behind means, medians and variance measures should be available. If there are restrictions on publicly sharing data—e.g. participant privacy or use of data from a third party—those must be specified.  Reviewer #2: Yes | We are glad that this was satisfactorily addressed in our first set of revisions |
| 5. Is the manuscript presented in an intelligible fashion and written in standard English?  PLOS ONE does not copyedit accepted manuscripts, so the language in submitted articles must be clear, correct, and unambiguous. Any typographical or grammatical errors should be corrected at revision, so please note any specific errors here.  Reviewer #2: Yes | We are glad that this was satisfactorily addressed in our first set of revisions |
| Reviewer #2: Summary of review The authors have done a commendable job incorporating the previous recommendations. I have additional recommendations to further strengthen the manuscript outlined below. | We appreciate the guidance of the reviewers towards improving our work. We further continued to address the additional comments as discussed below. |
| New comments after second review | Response from author |
| 1. There continue to be typos and English grammar problems throughout the manuscript. 2. Please use precise writing throughout your manuscript – several examples of imprecision are outlined below in the Intro paragraph, “which district”, “prevalence of what”? | **The entire manuscript has been reviewed with focus on clearing typos, grammar errors and precise writing.** |
| I’m struggling to understand how the health center survey fits in with the virologic suppression analysis. A starting point would be to describe the clinic/hospital level of each health center you surveyed. Is there a way to tie in the six health centers into the multivariable analysis of viral suppression? | **The health facility survey assessed health workers using structured questionnaires. The results from the survey were summarized using percentages and proportions and could not be integrated in the multivariable analysis.**  **Findings from the health facility were used to supplement the findings obtained from the multivariable analysis based on the patient characteristics. This has been rectified in the discussion section to clearly bring out the complete picture.** |
| 1. Page 3, Para 2 - This sentence is grammatically incorrect – please revise, “This mix of potential drivers of HIV transmission rises the need to contextualize more efficient methods of HIV prevention. 2. Page 3, Para 2 – “Basing on the assertion that undetectable viral load translates into no transmission of HIV (undetectable = untransmittable) [9-12], understanding the determinants of virological suppression could help in reducing the incidence of HIV in fishing communities [13, 14].” Change basing to based. 3. Overall, Paragraph 3 is confusing. I am not understanding the comparisons or what we are always discussing due to imprecise language. 4. Page 3, Para 3 – What are you comparing – what is this higher than? “The prevalence of HIV amongst the residents of the fishing communities is not known but is likely to be higher given the observed prevalence of HIV in the fishing communities along Lake Victoria [5].” | **These have been rectified mainly by improving sentence flow and grammar.** |
| 5. Page 3, Para 3 – clarify which district, “However, the general prevalence of HIV in the district based on 2020 program data in DHIS-2 was lower at 5.1% [16] as compared to the national prevalence of 6.2% reported in the UPHIA report of 2017 [6].” | **This has been rectified** |
| 6. Page 3, Para 3 – These percentages of non-suppression are among men with HIV (MWH) correct? As written, you state that this percentage is among all adult men. “In a study carried out in Uganda using national programme data in 2017, the prevalence of non-suppression among males was 13%. However, in Bulisa district, the prevalence of virological non-suppression amongst all adult males for the same period was much higher at 20% [17].” | **Indeed, this was confusing. This has been rectified.** |
| 7. This doesn’t make sense – you’re comparing the same thing, “Whereas there is the availability of program data about virological non-suppression for the districts with fishing communities in Uganda, little is known about the burden of virological non-suppression among the male-dominated fishing communities. | **Indeed, this was confusing. This has been rectified.** |
| Methods 1. Page 4, last sentence of “Study Setting” – what is the difference between facility-based management and facility-based improved management model, “40% on facility-based management, 6% on client-led ART delivery and 3% on the facility based improved management model.” | **Thanks for identifying this. Indeed, there was a mix of some terms. This has been rectified.** |
| 2. Under “Participant selection”, Is the “in charge” a person? Can you provide more detail (ie the nurse in charge)? “The HIV clinic in charge at each of the six health facilities serving patients living with HIV in the fishing communities was purposively selected for the face-to-face interviews to assess the contribution of the health facility to virological non-suppression.” | **This has been rectified.** |
| Page 6, “Data Collection Management”, I think you might mean 12 months from this study? As written it sounds like you mean the viral load was run up to 12 months after that blood vial’s collection. “An up-to-date viral load result was defined as a viral load result within 12 months from the time of taking off the blood sample” | **During the study, a viral load with in 12 months from the time of taking off the sample at the time of data collection was considered up to date.**  **Sentence further rectified to make it clearer**  **This is in line with the guidelines for HIV management in Uganda.** |
| 3. 4. Page 7, was pill count adherence based on participant report or did research staff count participant pills? | **Pill counting was done by the attending clinician. This has been rectified** |
| Results 1. Page 10, It would be helpful to clarify the corresponding age with each group. “However, they differed in their mean (SD) age (years), 40 (10.7) versus 35.9 (9.3), p=0.011.” | **The corresponding age was actually included however, it was probably confusing. This has been clarified. The figures in the brackets are Standard deviations.**  **40 (10.7) years versus 35.9 (9.3) years** |
| 2. Page 10, It makes more sense to compare the proportion of participants with viral non-suppression to those who did not did not participate in the study instead of comparing VL non-suppression among participants with any eligible participant. | **Yes, this sounds logical.**  **However, this comparison was made to gauge the comparability of the overall proportion of non-suppression among all the 413 patients that had a VL and the 379 that were successfully interviewed. The balance (413-379) was not included in the analysis of the factors and so we wanted to find out any form of bias.**  **The comparison of non-suppression with those that never participated was not possible because they never had a viral load for various reasons as highlighted in the flow chart in the manuscript.** |
| 3. Page 10, under “Demogrpahics” please define “mid-level health facility”. | **This has been defined as HC III or HC IV** |
| 4. Page 10, under “Demographics”, “About one-third reported having had 2 to 3 sexual partners in the previous 12 months and one-third never knew their partner’s HIV status.” Is partner knowledge status in reference to all partners? Any one partner? Please clarify. | **Knowledge status referred to all partners. This has been rectified by separating the two confusing sentences.** |
| 5. Page 13-14, under “Bivariable and multivariable analysis, you only discuss Bivariable analysis. I would potentially shorten the bivariable results and give more space to highlight the big takeaways from the multivariable analysis. | **A little confused with the comment.**  **We had excluded bivariable analysis narratives and concentrated on multivariable analysis.**  **We have now included both narratives for bivariable and multivariable narratives.** |
| 1. I suggest separating the discussion into cohesive paragraphs. I suggest reading other Discussion sections from high-impact journals for reference. A typical discussion paragraph will take a finding from your study that’s interesting to you, and put it in the context of broader research. You’ll typically try to compare this finding to research in similar settings or populations. Typically, you’ll want to describe the other studies a bit as your reader won’t necessarily have time to look at your references. You’ll then try to formulate a take-away, such as a suggestion or interpretation for how your study finding fits into the larger data. You don’t need to address every finding from your results section – only the findings you think are interesting. You begin to do this later in the discussion section. | **The entire discussion section has been re-written with your guidance.** |
| 2. I would try to better tie in the health center survey to the virologic nonsuppression outcome. As it stands these seem like two completely separate projects. Better framing in the discussion could help this issue. | **This has been rectified in the discussion section.** |
| Figures/Tables 1. Table 1. Under “number of sexual partners in previous 12 mo”, I suggest changing “only one” to “one”. | **This has been rectified.** |
| 2. Table 1. Under “Quality of clinical appointments and work”, what is the difference between “long enough” and “they are just fine”? These two fields do not seem exclusionary. | **“Long enough” means that the patient perceived the appointment duration to be of good duration to allow him/ or her to carry on duties without frequenting the facility**  **“Just fine” meant that the patient took a middle ground on the perception of the appointment duration. Not to short to disrupt work neither long enough to allow for sufficient time without going back for refills.** |
| 3. Table 2. I am confused by the column headings: “No – suppressed” and Yes-non-suppressed”. I would remove the No/Yes and simply write suppressed vs. non-suppressed. | **Indeed, this was confusing. This has been rectified** |
| 4. Table 2. It looks like you are trying to bold any p-values <0.05. If this is the case, you should also bold TB diagnosed in past 12 mo. | **This has been rectified** |
| 5. Table 3 took me a few minutes to comprehend. It might be visually easier to digest the information you’re trying to describe by having the six health centers at column headers, and then simply checkmark which health centers respond yes to each question | This has been done as guided and indeed the table is easier to understand. Thank you. |
