# Supplementary material for: Virological non-suppression among adult males attending HIV care services in the fishing communities in Bulisa district, Uganda
Source: PLoS One. 2023 Oct 19;18(10):e0293057. doi: 10.1371/journal.pone.0293057 (PMC10586650; doi:10.1371/journal.pone.0293057)
Supplement: Attachment — Submitted filename: Response to reviewers.docx [file pone.0293057.s017.docx]

Response

| **Comment** | **Response** |
| --- | --- |
| **Comments to the Author**  Reviewer #2: Overall The authors have done a commendable job incorporating the previous recommendations. I have additional recommendations to further strengthen the manuscript outlined below. | **We appreciate the support to make the manuscript better** |
| Introduction 1. Page 3, Paragraph 1: A prevalence is not a rate – please revise. | **This has been rectified as guided.** |
| 2. Page 4, Paragraph 1, Line 1: Clarify type of non-suppression (virologic) as done later in the sentence. | **This has been rectified as guided.** |
| Methods 1. Study setting: I recommend including the number of people living in this district/setting – this would put the number of people living with HIV into context. | **The population has been added using latest census results. The proportion of living with HIV in the district also included.** |
| 2. Page 7, Data collection and management: Please cite references for the sentences, The patients’ questionnaire was structured and designed based on characteristics common among residents of fishing communities and factors previously associated with virological non-suppression from other population segments” and “The Health worker’s questionnaire was structured and designed based on factors identified in previous qualitative and quantitative studies that focused on the quality of health care.” | **This has been rectified. However, the references had earlier been included for the particular sections/ characteristics used in designing the references.** |
| 3. Page 7, punctuation errors in the phrase: “adherence based on pill count by the attending clinician categorized as good (>95%), fair (80-95%) poor (<80%), fulfilment of clinical appointments; ”should be, “adherence based on pill count by the attending clinician categorized as good (>95%), fair (80-95%), poor (<80%); fulfilment of clinical appointments;” | **Punctuation error rectified** |
| 4. Page 10, The following sentence does not make sense, “Out of the 462 participants who fulfilled the eligibility criteria, 367 were excluded because they were non-residents”. Residing in the district is an eligibility criterion and above the authors state 462 participants met criteria. | **The confusion has been rectified. Sentence reconstruction and clarity done.** |
| 5. Page 11, the authors compare participants interviewed vs. those who did were not interviewed for several categories, but they compare participants interviewed to all participants for viral load. I recommend they consistently compare those interviewed vs. those who did not interview. | **The comparison of participants interviewed to all participants with viral load indeed created some confusion.**  **To avoid confusion, this was deleted to maintain interviewed vs non-interviewed.** |
| 6. How did the authors ask about HIV disclosure? Could participants state disclosure to multiple categories or were they to only choose one? Please clarify in the manuscript. | **Participants were requested to pick an option from the list. Not framing the questions to assess for multiple disclosure has been captured as a limitation to the study** |
| 7. How did the authors partner HIV status in regards to people with multiple partners? Please clarify in the manuscript. | **The question for partner HIV status never had no provision for multiple partners. This has been captured as a limitation.** |
| 8. Under quality of clinical appointments and work, please clarify the difference between, “long enough” and “they are just fine”. Please clarify in the manuscript. | **During the interviews, the statement in relation to appointments “they are just fine”, was used to mean “appointment intervals that were “neither long or short” To avoid confusion, this has been clarified and the wording explained under methodology. Quality of clinical appointments has been updated to “perceived length of clinic appointments”** |
| 9. Under viral load turnaround time, please clarify “can’t tell”. Does this mean the participant didn’t know or didn’t want to report this in the study? | **“Can’t tell me” meant “did not Know”. This has been updated** |
| Results 1. Please define CPR at first use. | **This has been defined under methodology.** |
| 2. It is difficult to think about prevalence ratios < 1. I recommend reversing your reference labels to have results where your prevalence ratios are all > 1. | **This has been rectified. It mainly affected results at multivariable analysis. Results at multivariable analysis had been rectified with earlier corrections.** |
| 3. Please revise this sentence as it is difficult to follow, “The prevalence of virological non-suppression amongst participants who received care from the only hospital in the district was 1.22 times (adj.PR=1.22, 95%CI: 1.08-1.37) and 0.61 (adj.PR=0.61, 95%CI: 0.57-0.66) amongst participants that sought treatment at health centre III levels compared to the prevalence of virological non-suppression amongst participants who received care at health Centre II level.” | **Sentence among those that were dropped to cut on the word count.** |
| 4. Overall you could cut down significantly on the word count of the Results section. Since this data is in the Tables, you should only write out the most important findings you wish to highlight. | **This has been rectified especially at multivariable analysis** |
| 5. Why wasn’t ARV regimen change or ARV line of therapy in the multivariable analysis since these seemed to significantly associated with virologic non-suppression? | **The mode of analysis mainly focused at finding the relationship between unique factors amongst patients’ resident in the fishing communities. So, these were prioritized. Since, we had limitations on the number of factors to consider (only 8), yet we had so many factors that were significant at bivariable analysis, we picked out a few that were significant but also biologically plausible. Similarly, those showed higher tendencies of collinearity were dropped.**  **The unique factors considered included:**   - **Hazardous use of alcohol** - **Frequency of moving between fish landing sites** - **No. Sexual partners in view of possible contribution to reinfections** - **Occupation**   **Facility factors**   - **Extending services closer to them given the nature of their work** - **Level of facility managing patients**   **Confounding factors**   - **Age** - **Diagnosis with TB (They work in groups, But, also TB has been associated with interrupting ART and even changing lines of treatment). This informed the dropping of some factors due to possible collinearity.**   **The factors included were carefully thought about** |
| 6. It appears that there are 9 variables in the multivariable model. Are the authors concerned about overfitting the model? Especially since participants without viral suppression were 84 (in regards to the 1/10 regression rule). | **These have been dropped to 8. ARV interruption was dropped and TB was maintained given the explanation above.** |
| 7. The data regarding the health facility factors does not seem relevant in this analysis since the authors do not tie this into the viral non-suppression in any way. I would recommend removing this and potentially it could be a short report. | **Not sure whether the comment has been understood.**  **However, Preparedness of health workers to serve people living with HIV and quality of services offered to people living with HIV was used mainly to beef up the explanation under discussion.**  **However, we agree that the health facility factors under logistic regression mainly focused on extending services to patients and level of health facility in the final. Dropping these, however would change the entire analysis.** |
| Discussion 1. In regards to limitations, the authors state, “An additional limitation could be the lack of back translation from the local language to English which could have led to a loss of meaning in the translation of some data.” If the authors and people doing the analysis understood and were fluent in the local language, then there is no need for English translation. | **This limitation has been dropped** |
